# Supplementary material for: Synthesis, Crystal Structure and Bioactivity of Phenazine-1-carboxylic Acylhydrazone Derivatives
Source: Molecules. 2021 Sep 1;26(17):5320. doi: 10.3390/molecules26175320 (PMC8434039; doi:10.3390/molecules26175320)

checkCIF/PLATON report

Structure factors have been supplied for datablock(s) ww

THIS REPORT IS FOR GUIDANCE ONLY. IF USED AS PART OF A REVIEW PROCEDURE FOR PUBLICATION, IT SHOULD NOT REPLACE THE EXPERTISE OF AN EXPERIENCED CRYSTALLOGRAPHIC REFEREE.

No syntax errors found. CIF dictionary Interpreting this report

Datablock: ww

```
Bond precision:  C-C = 0.0226 A          Wavelength=0.71073

Cell:           a=7.133(10)       b=9.190(13)      c=18.84(3)
                alpha=81.52(2)    beta=87.70(3)    gamma=74.49(3)
Temperature:    293 K

                Calculated                 Reported
Volume          1177(3)                    1177(3)
Space group     P -1                       P -1
Hall group      -P 1                       -P 1
Moiety formula  C23 H15 N5 O, C H Cl3      C23 H15 N5 O, C H Cl3
Sum formula     C24 H16 Cl3 N5 O           C24 H16 Cl3 N5 O
Mr              496.77                     496.77
Dx,g cm-3       1.402                      1.402
Z               2                          2
Mu (mm-1)       0.416                      0.416
F000            508.0                      508.0
F000'           509.04
h,k,lmax        8,10,22                    8,10,22
Nref            4150                       3329
Tmin,Tmax       0.942,0.963
Tmin'           0.940

Correction method= Not given

Data completeness= 0.802          Theta(max)= 24.999

R(reflections)= 0.2464( 1293)     wR2(reflections)= 0.6606( 3329)

S = 1.246                  Npar= 307
```

The following ALERTS were generated. Each ALERT has the format
test-name_ALERT_alert-type_alert-level.
Click on the hyperlinks for more details of the test.

Alert level A

```
PLAT029_ALERT_3_A _diffrn_measured_fraction_theta_full value Low .      0.802 Why?
PLAT082_ALERT_2_A High R1 Value ..................................       0.25 Report
PLAT084_ALERT_3_A High wR2 Value (i.e. > 0.25) ...................       0.66 Report
```

Alert level B

```
PLAT026_ALERT_3_B Ratio Observed / Unique Reflections (too) Low ..        39% Check
PLAT340_ALERT_3_B Low Bond Precision on  C-C Bonds ...............    0.02261 Ang.
PLAT911_ALERT_3_B Missing FCF Refl Between Thmin & STh/L=    0.595        822 Report
```

Alert level C

```
PLAT052_ALERT_1_C Info on Absorption Correction Method   Not Given     Please Do !
PLAT148_ALERT_3_C s.u. on the      a    - Axis is (Too) Large ....      0.010 Ang.
PLAT148_ALERT_3_C s.u. on the      b    - Axis is (Too) Large ....      0.013 Ang.
PLAT148_ALERT_3_C s.u. on the      c    - Axis is (Too) Large ....      0.030 Ang.
PLAT234_ALERT_4_C Large Hirshfeld Difference Cl1      --C47      .       0.21 Ang.
PLAT234_ALERT_4_C Large Hirshfeld Difference Cl1A     --C47      .       0.21 Ang.
PLAT241_ALERT_2_C High   'MainMol' Ueq as Compared to Neighbors of         C4 Check
PLAT244_ALERT_4_C Low    'Solvent' Ueq as Compared to Neighbors of        C47 Check
PLAT260_ALERT_2_C Large Average Ueq of Residue Including       Cl1      0.178 Check
PLAT336_ALERT_2_C Long Bond Distance for ..... C47      -Cl3A           2.000 Ang.
PLAT906_ALERT_3_C Large K Value in the Analysis of Variance ......    250.686 Check
PLAT906_ALERT_3_C Large K Value in the Analysis of Variance ......      5.526 Check
PLAT906_ALERT_3_C Large K Value in the Analysis of Variance ......     32.360 Check
PLAT906_ALERT_3_C Large K Value in the Analysis of Variance ......      2.983 Check
PLAT906_ALERT_3_C Large K Value in the Analysis of Variance ......      9.815 Check
PLAT906_ALERT_3_C Large K Value in the Analysis of Variance ......      5.236 Check
PLAT906_ALERT_3_C Large K Value in the Analysis of Variance ......      2.482 Check
PLAT976_ALERT_2_C Check Calcd Resid. Dens.  0.90A   From C47            -0.46 eA-3
```

Alert level G

```
PLAT007_ALERT_5_G Number of Unrefined Donor-H Atoms ..............          1 Report
PLAT072_ALERT_2_G SHELXL First  Parameter in WGHT  Unusually Large       0.38 Report
PLAT171_ALERT_4_G The CIF-Embedded .res File Contains EADP Records          3 Report
PLAT186_ALERT_4_G The CIF-Embedded .res File Contains ISOR Records          1 Report
PLAT199_ALERT_1_G Reported _cell_measurement_temperature ..... (K)        293 Check
PLAT200_ALERT_1_G Reported   _diffrn_ambient_temperature ..... (K)        293 Check
PLAT231_ALERT_4_G Hirshfeld Test (Solvent)   Cl2      --C47      .        9.0 s.u.
PLAT231_ALERT_4_G Hirshfeld Test (Solvent)   Cl2A     --C47      .        8.9 s.u.
PLAT231_ALERT_4_G Hirshfeld Test (Solvent)   Cl3      --C47      .        9.3 s.u.
PLAT231_ALERT_4_G Hirshfeld Test (Solvent)   Cl3A     --C47      .        9.1 s.u.
PLAT300_ALERT_4_G Atom Site Occupancy of Cl1        Constrained at        0.5 Check
PLAT300_ALERT_4_G Atom Site Occupancy of Cl1A       Constrained at        0.5 Check
PLAT300_ALERT_4_G Atom Site Occupancy of Cl2        Constrained at        0.5 Check
PLAT300_ALERT_4_G Atom Site Occupancy of Cl2A       Constrained at        0.5 Check
PLAT300_ALERT_4_G Atom Site Occupancy of Cl3        Constrained at        0.5 Check
PLAT300_ALERT_4_G Atom Site Occupancy of Cl3A       Constrained at        0.5 Check
PLAT302_ALERT_4_G Anion/Solvent/Minor-Residue Disorder (Resd  2  )        75% Note
PLAT333_ALERT_2_G Large Aver C6-Ring C-C Dist C12      -C17      .       1.42 Ang.
PLAT779_ALERT_4_G Suspect or Irrelevant (Bond) Angle(s) in CIF ...      38.00 Deg.
              CL1A -C47  -CL1     1_555   1_555   1_555 ......     #  100 Check
PLAT860_ALERT_3_G Number of Least-Squares Restraints .............        216 Note
PLAT909_ALERT_3_G Percentage of I>2sig(I) Data at Theta(Max) Still        43% Note
PLAT933_ALERT_2_G Number of OMIT Records in Embedded .res File ...          1 Note
PLAT941_ALERT_3_G Average HKL Measurement Multiplicity ...........        1.2 Low
PLAT961_ALERT_5_G Dataset Contains no Negative Intensities .......     Please Check
PLAT978_ALERT_2_G Number C-C Bonds with Positive Residual Density.          0 Info
```

```
  3 ALERT level A = Most likely a serious problem - resolve or explain
  3 ALERT level B = A potentially serious problem, consider carefully
 18 ALERT level C = Check. Ensure it is not caused by an omission or oversight
 25 ALERT level G = General information/check it is not something unexpected

  3 ALERT type 1 CIF construction/syntax error, inconsistent or missing data
  9 ALERT type 2 Indicator that the structure model may be wrong or deficient
 18 ALERT type 3 Indicator that the structure quality may be low
 17 ALERT type 4 Improvement, methodology, query or suggestion
  2 ALERT type 5 Informative message, check
```

It is advisable to attempt to resolve as many as possible of the alerts in all categories. Often the minor alerts point to easily fixed oversights, errors and omissions in your CIF or refinement strategy, so attention to these fine details can be worthwhile. In order to resolve some of the more serious problems it may be necessary to carry out additional measurements or structure refinements. However, the purpose of your study may justify the reported deviations and the more serious of these should normally be commented upon in the discussion or experimental section of a paper or in the "special_details" fields of the CIF. checkCIF was carefully designed to identify outliers and unusual parameters, but every test has its limitations and alerts that are not important in a particular case may appear. Conversely, the absence of alerts does not guarantee there are no aspects of the results needing attention. It is up to the individual to critically assess their own results and, if necessary, seek expert advice.

Publication of your CIF in IUCr journals

A basic structural check has been run on your CIF. These basic checks will be run on all CIFs submitted for publication in IUCr journals (*Acta Crystallographica*, *Journal of Applied Crystallography*, *Journal of Synchrotron Radiation*); however, if you intend to submit to *Acta Crystallographica Section C* or *E* or *IUCrData*, you should make sure that full publication checks are run on the final version of your CIF prior to submission.

Publication of your CIF in other journals

Please refer to the *Notes for Authors* of the relevant journal for any special instructions relating to CIF submission.

PLATON version of 13/07/2021; check.def file version of 13/07/2021

Datablock ww - ellipsoid plot

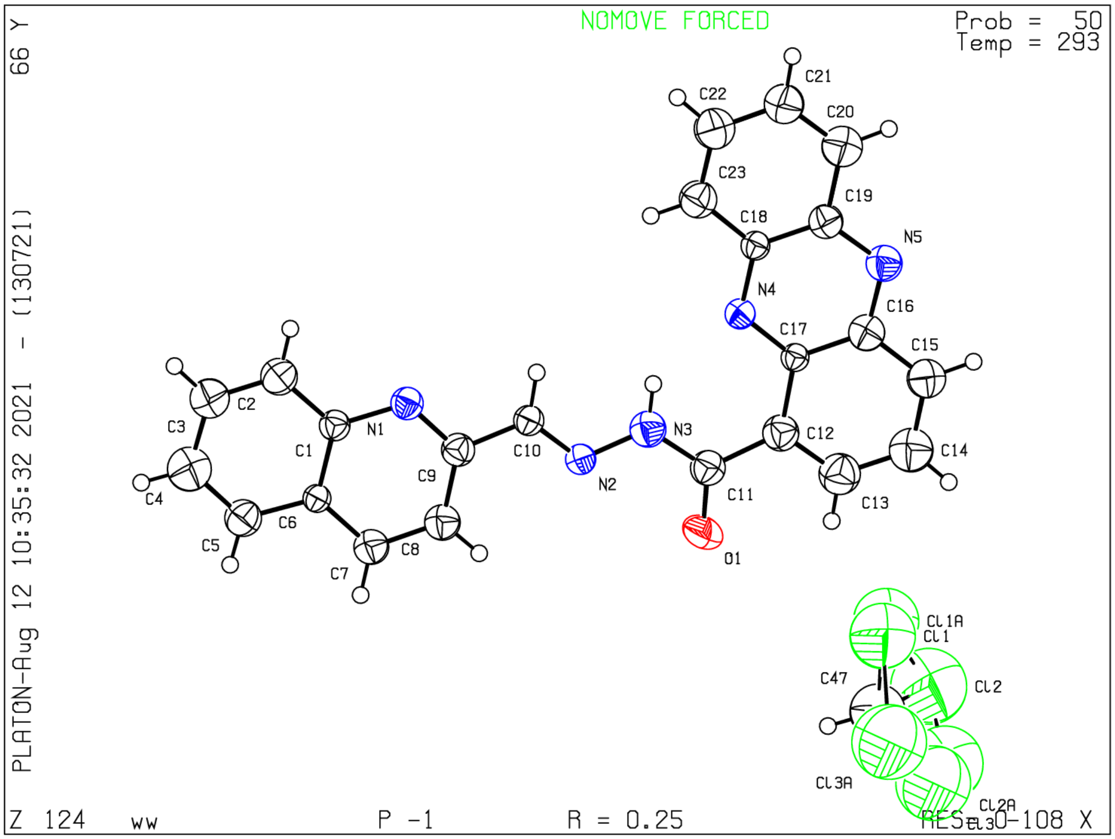

Supplement: Supplementary file 1 [file molecules-26-05320-s001.zip › cif and checkcif/cif and checkcif/3a.pdf]
