# Supplementary material for: Synthesis, Crystal Structure and Bioactivity of Phenazine-1-carboxylic Acylhydrazone Derivatives
Source: Molecules. 2021 Sep 1;26(17):5320. doi: 10.3390/molecules26175320 (PMC8434039; doi:10.3390/molecules26175320)

checkCIF/PLATON report

Structure factors have been supplied for datablock(s) I

THIS REPORT IS FOR GUIDANCE ONLY. IF USED AS PART OF A REVIEW PROCEDURE FOR PUBLICATION, IT SHOULD NOT REPLACE THE EXPERTISE OF AN EXPERIENCED CRYSTALLOGRAPHIC REFEREE.

No syntax errors found. CIF dictionary Interpreting this report

Datablock: I

```
Bond precision:  C-C = 0.0053 A          Wavelength=0.71073

Cell:            a=7.2472(8)     b=5.1293(5)         c=20.804(2)
                 alpha=90        beta=95.915(3)      gamma=90
Temperature:     293 K

                Calculated                Reported
Volume          769.23(14)                769.22(14)
Space group     P n                       P 1 n 1
Hall group      P -2yac                   P -2yac
Moiety formula  C19 H13 N5 O              C19 H13 N5 O
Sum formula     C19 H13 N5 O              C19 H13 N5 O
Mr              327.34                    327.34
Dx,g cm-3       1.413                     1.413
Z               2                         2
Mu (mm-1)       0.093                     0.093
F000            340.0                     340.0
F000'           340.12
h,k,lmax        8,6,24                    8,6,24
Nref            2654[ 1330]               2159
Tmin,Tmax       0.981,0.983               0.975,0.979
Tmin'           0.981

Correction method= # Reported T Limits: Tmin=0.975 Tmax=0.979
AbsCorr = NONE

Data completeness= 1.62/0.81      Theta(max)= 24.806

R(reflections)= 0.0365( 1834)     wR2(reflections)= 0.0859( 2159)

S = 1.030                  Npar= 226
```

The following ALERTS were generated. Each ALERT has the format
test-name_ALERT_alert-type_alert-level.
Click on the hyperlinks for more details of the test.

Alert level C

```
STRVA01_ALERT_4_C           Flack parameter is too small
           From the CIF: _refine_ls_abs_structure_Flack   -2.800
           From the CIF: _refine_ls_abs_structure_Flack_su    1.000
PLAT089_ALERT_3_C Poor Data / Parameter Ratio (Zmax < 18) ........       5.85 Note
PLAT340_ALERT_3_C Low Bond Precision on  C-C Bonds ...............    0.00528 Ang.
PLAT911_ALERT_3_C Missing FCF Refl Between Thmin & STh/L=    0.590          8 Report
```

Alert level G

```
PLAT007_ALERT_5_G Number of Unrefined Donor-H Atoms ..............          1 Report
PLAT032_ALERT_4_G Std. Uncertainty on Flack Parameter Value High .      1.000 Report
PLAT066_ALERT_1_G Predicted and Reported Tmin&Tmax Range Identical          ? Check
PLAT199_ALERT_1_G Reported _cell_measurement_temperature ..... (K)        293 Check
PLAT200_ALERT_1_G Reported   _diffrn_ambient_temperature ..... (K)        293 Check
PLAT720_ALERT_4_G Number of Unusual/Non-Standard Labels ..........          1 Note
PLAT883_ALERT_1_G No Info/Value for _atom_sites_solution_primary .     Please Do !
PLAT909_ALERT_3_G Percentage of I>2sig(I) Data at Theta(Max) Still        50% Note
PLAT915_ALERT_3_G No Flack x Check Done: Low Friedel Pair Coverage         63 %
PLAT916_ALERT_2_G Hooft y and Flack x Parameter Values Differ by .       1.40 Check
PLAT941_ALERT_3_G Average HKL Measurement Multiplicity ...........        3.5 Low
PLAT978_ALERT_2_G Number C-C Bonds with Positive Residual Density.          0 Info
```

---

   0 **ALERT level A** = Most likely a serious problem - resolve or explain
   0 **ALERT level B** = A potentially serious problem, consider carefully
   4 **ALERT level C** = Check. Ensure it is not caused by an omission or oversight
  12 **ALERT level G** = General information/check it is not something unexpected

   4 ALERT type 1 CIF construction/syntax error, inconsistent or missing data
   2 ALERT type 2 Indicator that the structure model may be wrong or deficient
   6 ALERT type 3 Indicator that the structure quality may be low
   3 ALERT type 4 Improvement, methodology, query or suggestion
   1 ALERT type 5 Informative message, check

---

checkCIF publication errors

Alert level A

```
PUBL004_ALERT_1_A  The contact author's name and address are missing,
            _publ_contact_author_name and _publ_contact_author_address.
PUBL005_ALERT_1_A  _publ_contact_author_email, _publ_contact_author_fax and
            _publ_contact_author_phone are all missing.
            At least one of these should be present.
PUBL006_ALERT_1_A  _publ_requested_journal is missing
            e.g. 'Acta Crystallographica Section C'
PUBL008_ALERT_1_A  _publ_section_title is missing. Title of paper.
PUBL009_ALERT_1_A  _publ_author_name is missing. List of author(s) name(s).
PUBL010_ALERT_1_A  _publ_author_address is missing. Author(s) address(es).
PUBL012_ALERT_1_A  _publ_section_abstract is missing.
            Abstract of paper in English.
```

---

   7 **ALERT level A** = Data missing that is essential or data in wrong format
   0 **ALERT level G** = General alerts. Data that may be required is missing

Publication of your CIF

You should attempt to resolve as many as possible of the alerts in all categories. Often the minor alerts point to easily fixed oversights, errors and omissions in your CIF or refinement strategy, so attention to these fine details can be worthwhile. In order to resolve some of the more serious problems it may be necessary to carry out additional measurements or structure refinements. However, the nature of your study may justify the reported deviations from journal submission requirements and the more serious of these should be commented upon in the discussion or experimental section of a paper or in the "special_details" fields of the CIF. *checkCIF* was carefully designed to identify outliers and unusual parameters, but every test has its limitations and alerts that are not important in a particular case may appear. Conversely, the absence of alerts does not guarantee there are no aspects of the results needing attention. It is up to the individual to critically assess their own results and, if necessary, seek expert advice.

If level A alerts remain, which you believe to be justified deviations, and you intend to submit this CIF for publication in a journal, you should additionally insert an explanation in your CIF using the Validation Reply Form (VRF) below. This will allow your explanation to be considered as part of the review process.

Validation response form

Please find below a validation response form (VRF) that can be filled in and pasted into your CIF.

```
# start Validation Reply Form
_vrf_PUBL004_GLOBAL
;
PROBLEM: The contact author’s name and address are missing,
RESPONSE: ...
;
_vrf_PUBL005_GLOBAL
;
PROBLEM: _publ_contact_author_email, _publ_contact_author_fax and
RESPONSE: ...
;
_vrf_PUBL006_GLOBAL
;
PROBLEM: _publ_requested_journal is missing
RESPONSE: ...
;
_vrf_PUBL008_GLOBAL
;
PROBLEM: _publ_section_title is missing. Title of paper.
RESPONSE: ...
;
_vrf_PUBL009_GLOBAL
;
PROBLEM: _publ_author_name is missing. List of author(s) name(s).
RESPONSE: ...
;
_vrf_PUBL010_GLOBAL
;
PROBLEM: _publ_author_address is missing. Author(s) address(es).
RESPONSE: ...
;
_vrf_PUBL012_GLOBAL
;
```

```
PROBLEM: _publ_section_abstract is missing.
RESPONSE: ...
;
# end Validation Reply Form
```

If you wish to submit your CIF for publication in Acta Crystallographica Section C or E, you should upload your CIF via the web. If you wish to submit your CIF for publication in IUCrData you should upload your CIF via the web. If your CIF is to form part of a submission to another IUCr journal, you will be asked, either during electronic submission or by the Co-editor handling your paper, to upload your CIF via our web site.

PLATON version of 18/09/2020; check.def file version of 20/08/2020

Datablock I - ellipsoid plot

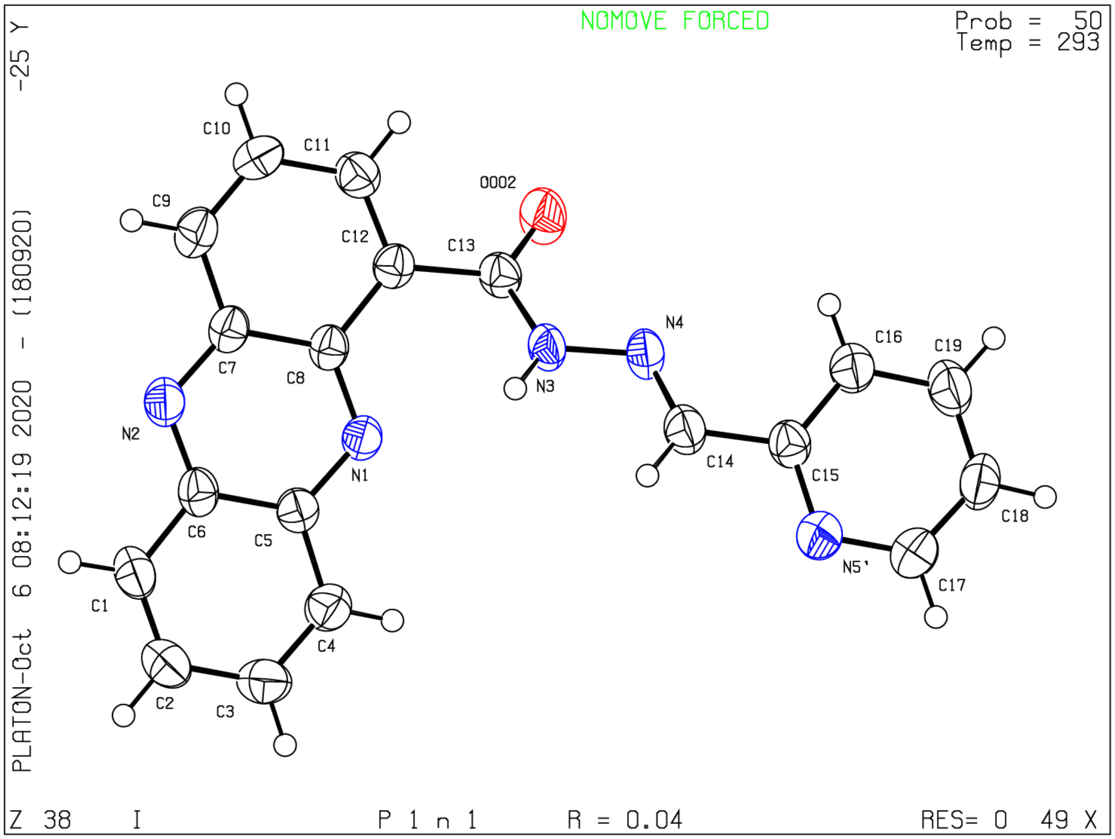

Supplement: Supplementary file 1 [file molecules-26-05320-s001.zip › cif and checkcif/cif and checkcif/3g.pdf]
