# Supplementary material for: Synthesis, Crystal Structure and Bioactivity of Phenazine-1-carboxylic Acylhydrazone Derivatives
Source: Molecules. 2021 Sep 1;26(17):5320. doi: 10.3390/molecules26175320 (PMC8434039; doi:10.3390/molecules26175320)
Supplement: Supplementary file 1 [file molecules-26-05320-s001.zip › cif and checkcif/cif and checkcif/a_cifreport.html]

checkCIF/PLATON page 2


# checkCIF (basic structural check) running

---

  
*Checking for embedded fcf data in CIF ...*
  
*Found embedded fcf data in CIF. Extracting fcf data from uploaded CIF, please wait*
**.**
**.**
**.**

# checkCIF/PLATON (basic structural check)

---

Structure factors have been supplied for datablock(s) a

THIS REPORT IS FOR GUIDANCE ONLY. IF USED AS PART OF A REVIEW PROCEDURE FOR PUBLICATION, IT SHOULD NOT REPLACE THE EXPERTISE OF AN EXPERIENCED CRYSTALLOGRAPHIC REFEREE.

```
No syntax errors found.                               CIF dictionary  
Please wait while processing ....                     Interpreting this report
```

Structure factor report  
