# Supplementary material for: Synthesis, Crystal Structure and Bioactivity of Phenazine-1-carboxylic Acylhydrazone Derivatives
Source: Molecules. 2021 Sep 1;26(17):5320. doi: 10.3390/molecules26175320 (PMC8434039; doi:10.3390/molecules26175320)
Supplement: Supplementary file 1 [file molecules-26-05320-s001.zip › cif and checkcif/cif and checkcif/a_tables.html]

a


# a

Table 1 Crystal data and structure refinement for a.

| Identification code | a |
| Empirical formula | C24H16N4O2 |
| Formula weight | 392.41 |
| Temperature/K | 293(2) |
| Crystal system | monoclinic |
| Space group | P21/n |
| a/Å | 7.6260(17) |
| b/Å | 13.156(3) |
| c/Å | 18.946(4) |
| α/° | 90 |
| β/° | 98.776(7) |
| γ/° | 90 |
| Volume/Å3 | 1878.6(7) |
| Z | 4 |
| ρcalcg/cm3 | 1.387 |
| μ/mm‑1 | 0.091 |
| F(000) | 816.0 |
| Crystal size/mm3 | 0.23 × 0.21 × 0.16 |
| Radiation | MoKα (λ = 0.71073) |
| 2Θ range for data collection/° | 3.784 to 49.996 |
| Index ranges | -9 ≤ h ≤ 9, -15 ≤ k ≤ 15, -19 ≤ l ≤ 22 |
| Reflections collected | 11916 |
| Independent reflections | 3302 [Rint = 0.0505, Rsigma = 0.0626] |
| Data/restraints/parameters | 3302/0/271 |
| Goodness-of-fit on F2 | 1.039 |
| Final R indexes [I>=2σ (I)] | R1 = 0.0617, wR2 = 0.1697 |
| Final R indexes [all data] | R1 = 0.1411, wR2 = 0.2189 |
| Largest diff. peak/hole / e Å-3 | 0.27/-0.16 |

Table 2 Fractional Atomic Coordinates (×104) and Equivalent Isotropic Displacement Parameters (Å2×103) for a. Ueq is defined as 1/3 of of the trace of the orthogonalised UIJ tensor.

| Atom | *x* | *y* | *z* | U(eq) |
| C1 | 10075(5) | 1976(3) | 5920(2) | 69.9(10) |
| C2 | 9834(6) | 1547(3) | 6542(2) | 85.2(13) |
| C3 | 8693(6) | 696(3) | 6553(3) | 89.8(13) |
| C4 | 7886(6) | 281(3) | 5943(2) | 79.7(12) |
| C5 | 8143(5) | 679(3) | 5267(2) | 67.3(10) |
| C6 | 9255(4) | 1568(2) | 5263(2) | 57.4(9) |
| C7 | 8668(4) | 1604(2) | 4030(2) | 56.5(9) |
| C8 | 7613(5) | 693(3) | 4039(2) | 63.9(10) |
| C9 | 8829(5) | 2052(3) | 3359(2) | 61.3(9) |
| C10 | 8036(5) | 1584(3) | 2750(2) | 78.2(12) |
| C11 | 7042(6) | 671(3) | 2767(3) | 86.6(13) |
| C12 | 6825(5) | 254(3) | 3390(3) | 81.1(12) |
| C13 | 9733(5) | 3053(3) | 3259(2) | 68.5(11) |
| C14 | 11984(5) | 4838(2) | 4426(2) | 60.9(10) |
| C15 | 13003(4) | 5784(2) | 4458(2) | 58.5(9) |
| C16 | 13255(5) | 6267(3) | 3840(2) | 72.4(11) |
| C17 | 14309(6) | 7164(3) | 3854(3) | 84.4(13) |
| C18 | 15067(5) | 7533(3) | 4500(3) | 87.0(14) |
| C19 | 14854(5) | 7086(3) | 5152(3) | 69.7(11) |
| C20 | 15631(5) | 7479(3) | 5818(3) | 87.6(14) |
| C21 | 15421(6) | 7033(3) | 6444(3) | 92.0(14) |
| C22 | 14394(6) | 6164(3) | 6420(2) | 86.5(13) |
| C23 | 13607(5) | 5763(3) | 5794(2) | 67.7(10) |
| C24 | 13786(4) | 6189(2) | 5134(2) | 58.7(9) |
| N1 | 7366(4) | 258(2) | 4660(2) | 72.6(9) |
| N2 | 9475(3) | 2007.1(19) | 4644.0(16) | 58.0(8) |
| N3 | 10414(4) | 3542(2) | 3859.9(16) | 65.0(8) |
| N4 | 11248(4) | 4466(2) | 3823.7(17) | 65.7(9) |
| O1 | 9807(4) | 3392(2) | 2665.8(15) | 100.3(10) |
| O2 | 12584(4) | 5931(2) | 3176.8(15) | 94.0(9) |

Table 3 Anisotropic Displacement Parameters (Å2×103) for a. The Anisotropic displacement factor exponent takes the form: -2π2[h2a\*2U11+2hka\*b\*U12+…].

| Atom | U11 | U22 | U33 | U23 | U13 | U12 |
| C1 | 71(3) | 62(2) | 76(3) | 6(2) | 9(2) | -2(2) |
| C2 | 94(3) | 79(3) | 81(3) | 6(2) | 10(3) | -5(3) |
| C3 | 102(3) | 75(3) | 94(3) | 16(3) | 22(3) | 1(3) |
| C4 | 90(3) | 49(2) | 103(3) | 11(2) | 26(3) | -5(2) |
| C5 | 66(3) | 42.6(19) | 96(3) | 3(2) | 21(2) | 8.0(19) |
| C6 | 55(2) | 42.7(19) | 76(3) | 4.5(19) | 13.8(19) | 4.8(17) |
| C7 | 46(2) | 48(2) | 75(3) | -4(2) | 8.0(19) | 4.6(17) |
| C8 | 62(2) | 51(2) | 78(3) | -10(2) | 8(2) | 5.7(19) |
| C9 | 59(2) | 51(2) | 72(2) | -8(2) | 3(2) | 9.5(18) |
| C10 | 78(3) | 75(3) | 81(3) | -7(2) | 10(2) | 11(2) |
| C11 | 80(3) | 80(3) | 94(3) | -27(3) | -2(3) | -5(2) |
| C12 | 76(3) | 64(2) | 102(3) | -22(3) | 11(3) | -5(2) |
| C13 | 77(3) | 63(2) | 65(2) | 0(2) | 10(2) | 13(2) |
| C14 | 63(2) | 48(2) | 76(3) | 5.4(19) | 23(2) | 5.3(18) |
| C15 | 52(2) | 42.0(19) | 86(3) | 13(2) | 23(2) | 7.3(16) |
| C16 | 72(3) | 59(2) | 89(3) | 9(2) | 23(2) | 13(2) |
| C17 | 87(3) | 57(2) | 119(4) | 28(3) | 45(3) | 8(2) |
| C18 | 63(3) | 50(2) | 153(4) | 7(3) | 31(3) | 5(2) |
| C19 | 55(2) | 42(2) | 116(4) | -5(2) | 25(2) | 6.1(18) |
| C20 | 61(3) | 51(2) | 152(4) | -19(3) | 22(3) | -1(2) |
| C21 | 77(3) | 75(3) | 125(4) | -23(3) | 18(3) | 0(3) |
| C22 | 76(3) | 89(3) | 94(3) | -14(3) | 14(3) | 15(3) |
| C23 | 60(3) | 56(2) | 89(3) | -7(2) | 19(2) | 5.6(18) |
| C24 | 48(2) | 42.5(19) | 88(3) | -3(2) | 20(2) | 10.0(17) |
| N1 | 67(2) | 46.7(17) | 105(3) | -6.9(19) | 16(2) | -3.3(15) |
| N2 | 50.1(17) | 49.0(16) | 74(2) | 0.3(16) | 8.4(15) | 2.7(14) |
| N3 | 68(2) | 58.6(18) | 70(2) | 1.8(16) | 15.7(16) | -8.3(16) |
| N4 | 68(2) | 50.9(18) | 79(2) | 3.8(17) | 16.2(17) | 3.5(16) |
| O1 | 149(3) | 74.8(19) | 76(2) | 4.6(16) | 11.0(19) | 1.7(18) |
| O2 | 108(2) | 91(2) | 87(2) | 17.4(18) | 29.6(18) | 1.0(18) |

Table 4 Bond Lengths for a.

| Atom | Atom | Length/Å |  | Atom | Atom | Length/Å |
| C1 | C2 | 1.345(5) |  | C13 | N3 | 1.342(4) |
| C1 | C6 | 1.411(5) |  | C13 | O1 | 1.218(4) |
| C2 | C3 | 1.420(5) |  | C14 | C15 | 1.464(5) |
| C3 | C4 | 1.340(5) |  | C14 | N4 | 1.289(4) |
| C4 | C5 | 1.425(5) |  | C15 | C16 | 1.370(5) |
| C5 | C6 | 1.445(5) |  | C15 | C24 | 1.430(5) |
| C5 | N1 | 1.332(4) |  | C16 | C17 | 1.426(5) |
| C6 | N2 | 1.340(4) |  | C16 | O2 | 1.357(4) |
| C7 | C8 | 1.445(5) |  | C17 | C18 | 1.361(5) |
| C7 | C9 | 1.424(5) |  | C18 | C19 | 1.400(5) |
| C7 | N2 | 1.339(4) |  | C19 | C20 | 1.408(5) |
| C8 | C12 | 1.408(5) |  | C19 | C24 | 1.431(5) |
| C8 | N1 | 1.347(4) |  | C20 | C21 | 1.354(6) |
| C9 | C10 | 1.365(5) |  | C21 | C22 | 1.383(6) |
| C9 | C13 | 1.511(5) |  | C22 | C23 | 1.352(5) |
| C10 | C11 | 1.423(5) |  | C23 | C24 | 1.397(5) |
| C11 | C12 | 1.335(5) |  | N3 | N4 | 1.377(4) |

Table 5 Bond Angles for a.

| Atom | Atom | Atom | Angle/˚ |  | Atom | Atom | Atom | Angle/˚ |
| C2 | C1 | C6 | 120.8(4) |  | O1 | C13 | N3 | 122.7(4) |
| C1 | C2 | C3 | 120.7(4) |  | N4 | C14 | C15 | 121.1(3) |
| C4 | C3 | C2 | 120.7(4) |  | C16 | C15 | C14 | 120.1(4) |
| C3 | C4 | C5 | 121.2(4) |  | C16 | C15 | C24 | 119.9(3) |
| C4 | C5 | C6 | 117.6(4) |  | C24 | C15 | C14 | 120.0(3) |
| N1 | C5 | C4 | 121.3(4) |  | C15 | C16 | C17 | 121.3(4) |
| N1 | C5 | C6 | 121.1(4) |  | O2 | C16 | C15 | 123.9(4) |
| C1 | C6 | C5 | 118.9(4) |  | O2 | C16 | C17 | 114.7(4) |
| N2 | C6 | C1 | 120.7(3) |  | C18 | C17 | C16 | 118.2(4) |
| N2 | C6 | C5 | 120.4(3) |  | C17 | C18 | C19 | 123.6(4) |
| C9 | C7 | C8 | 118.6(3) |  | C18 | C19 | C20 | 123.1(4) |
| N2 | C7 | C8 | 120.1(3) |  | C18 | C19 | C24 | 117.9(4) |
| N2 | C7 | C9 | 121.4(3) |  | C20 | C19 | C24 | 119.0(4) |
| C12 | C8 | C7 | 119.6(4) |  | C21 | C20 | C19 | 122.4(4) |
| N1 | C8 | C7 | 121.1(3) |  | C20 | C21 | C22 | 118.2(5) |
| N1 | C8 | C12 | 119.3(4) |  | C23 | C22 | C21 | 121.6(5) |
| C7 | C9 | C13 | 125.2(3) |  | C22 | C23 | C24 | 122.6(4) |
| C10 | C9 | C7 | 118.7(4) |  | C15 | C24 | C19 | 119.1(4) |
| C10 | C9 | C13 | 116.0(4) |  | C23 | C24 | C15 | 124.6(3) |
| C9 | C10 | C11 | 122.0(4) |  | C23 | C24 | C19 | 116.3(4) |
| C12 | C11 | C10 | 120.4(4) |  | C5 | N1 | C8 | 118.3(3) |
| C11 | C12 | C8 | 120.6(4) |  | C7 | N2 | C6 | 119.1(3) |
| N3 | C13 | C9 | 115.9(3) |  | C13 | N3 | N4 | 120.2(3) |
| O1 | C13 | C9 | 121.4(4) |  | C14 | N4 | N3 | 115.6(3) |

Table 6 Hydrogen Atom Coordinates (Å×104) and Isotropic Displacement Parameters (Å2×103) for a.

| Atom | *x* | *y* | *z* | U(eq) |
| H1 | 10791 | 2549 | 5923 | 84 |
| H2 | 10420 | 1810 | 6969 | 102 |
| H3 | 8505 | 424 | 6989 | 108 |
| H4 | 7146 | -277 | 5961 | 96 |
| H10 | 8150 | 1870 | 2311 | 94 |
| H11 | 6540 | 364 | 2342 | 104 |
| H12 | 6148 | -333 | 3396 | 97 |
| H14 | 11867 | 4499 | 4847 | 73 |
| H17 | 14474 | 7488 | 3433 | 101 |
| H18 | 15764 | 8114 | 4509 | 104 |
| H20 | 16313 | 8066 | 5828 | 105 |
| H21 | 15952 | 7304 | 6877 | 110 |
| H22 | 14241 | 5847 | 6845 | 104 |
| H23 | 12920 | 5181 | 5804 | 81 |
| H3A | 10330 | 3279 | 4269 | 78 |
| H2A | 11747 | 5548 | 3202 | 141 |

a


# a

Table 1 Crystal data and structure refinement for a.

| Identification code | a |
| Empirical formula | C24H16N4O2 |
| Formula weight | 392.41 |
| Temperature/K | 293(2) |
| Crystal system | monoclinic |
| Space group | P21/n |
| a/Å | 7.6260(17) |
| b/Å | 13.156(3) |
| c/Å | 18.946(4) |
| α/° | 90 |
| β/° | 98.776(7) |
| γ/° | 90 |
| Volume/Å3 | 1878.6(7) |
| Z | 4 |
| ρcalcg/cm3 | 1.387 |
| μ/mm‑1 | 0.091 |
| F(000) | 816.0 |
| Crystal size/mm3 | 0.23 × 0.21 × 0.16 |
| Radiation | MoKα (λ = 0.71073) |
| 2Θ range for data collection/° | 3.784 to 49.996 |
| Index ranges | -9 ≤ h ≤ 9, -15 ≤ k ≤ 15, -19 ≤ l ≤ 22 |
| Reflections collected | 11916 |
| Independent reflections | 3302 [Rint = 0.0505, Rsigma = 0.0626] |
| Data/restraints/parameters | 3302/0/271 |
| Goodness-of-fit on F2 | 1.039 |
| Final R indexes [I>=2σ (I)] | R1 = 0.0617, wR2 = 0.1697 |
| Final R indexes [all data] | R1 = 0.1411, wR2 = 0.2189 |
| Largest diff. peak/hole / e Å-3 | 0.27/-0.16 |
