# Supplementary material for: Synthesis, Crystal Structure and Bioactivity of Phenazine-1-carboxylic Acylhydrazone Derivatives
Source: Molecules. 2021 Sep 1;26(17):5320. doi: 10.3390/molecules26175320 (PMC8434039; doi:10.3390/molecules26175320)
Supplement: Supplementary file 1 [file molecules-26-05320-s001.zip › molecules-SI.pdf]

Supporting Information

Synthesis, Crystal Structure and Bioactivity of Phenazine-1-carboxylic Acylhydrazone Derivatives

Shou-ting Wu [1,2], Xi Liang [1], Fang Luo [2], Hua Liu [2], Ling-yi Shen [1], Xian-jiong Yang [1,2], Ya-li Huang [1,2], Hong Xu [1,2], NingWu [1,2,]* and Qi-long Zhang[1,2,]*, Carl Redshaw [3]

1 Department of Basic Medical Sciences and Research Center for Molecular Medical Engineering, Guizhou Medical University, Guiyang 550004, China; wushouting1996@126.com (S.-T.W.); xuhong@gmc.edu.cn (H.X.); luo2020110110128@126.com(F.L.); liuhuaa008@126.com(H.L.); liangxi_15@163.com(X.L.)

2 Department of Biology & Engineering, Guizhou Medical University, Guiyang 550004, China; shen-ly@stumail.nwu.edu.cn (L.-Y.S.); ylh6401@gmc.edu.cn (Y.-L.H.); yangxianjiong@126.com (X.-J.Y.)

3 Department of Chemistry, University of Hull, Cottingham Road, Hull, Yorkshire HU6 7RX, UK; c.redshaw@hull.ac.uk

* Correspondence: wuning@gmc.edu.cn (N.W.); sciqlzhang@gmc.edu.cn; Fax: +86-0851-88174017 (Q.-L.Z.)

SUPPLEMENTARY MATERIAL

Contents

NMR of compounds 3a~3j S1-S10

HRMS of compounds 3a~3j S10-S14

Crystal data and structure refinement for the compound 3a~3c Table S1

Crystal data and structure refinement for the compound 3e~3g Table S2

Crystal data and structure refinement for the compound 3h~3k Table S3

Hydrogen bond parameters [Å and °] in the crystal structure of compounds 3a-3j Table S4

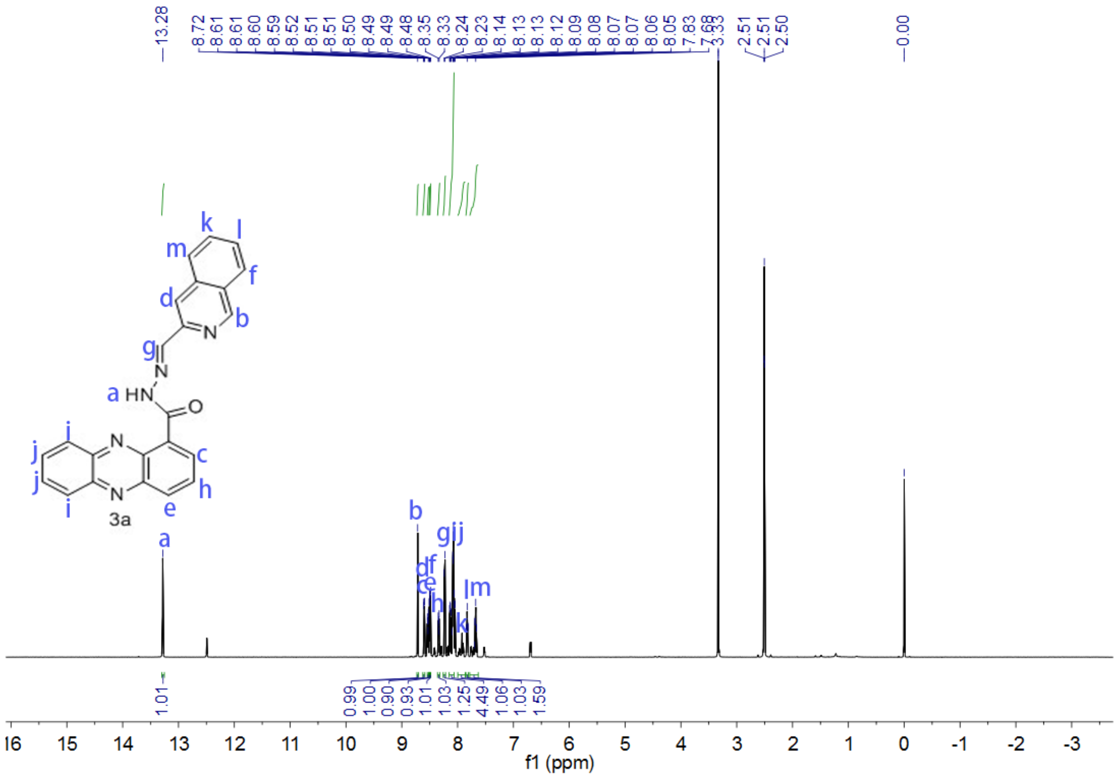

Figure S1. ^{1}H NMR of compound **3a**

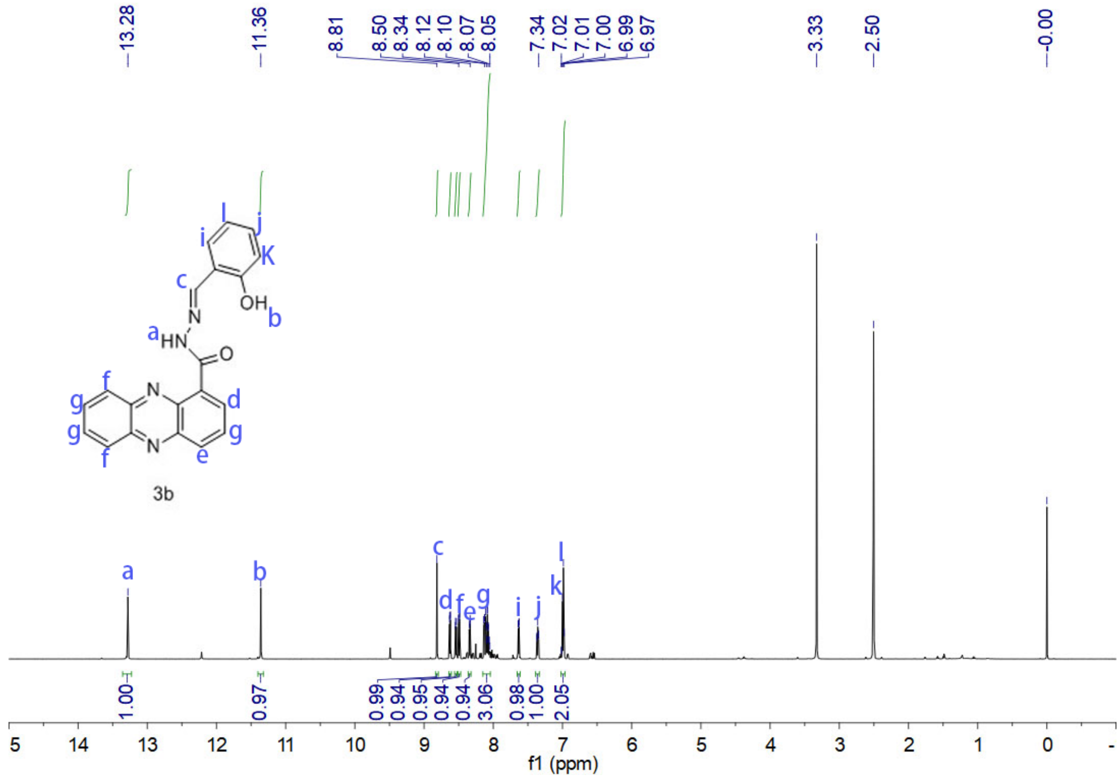

Figure S2. ^{1}H NMR of compound 3b

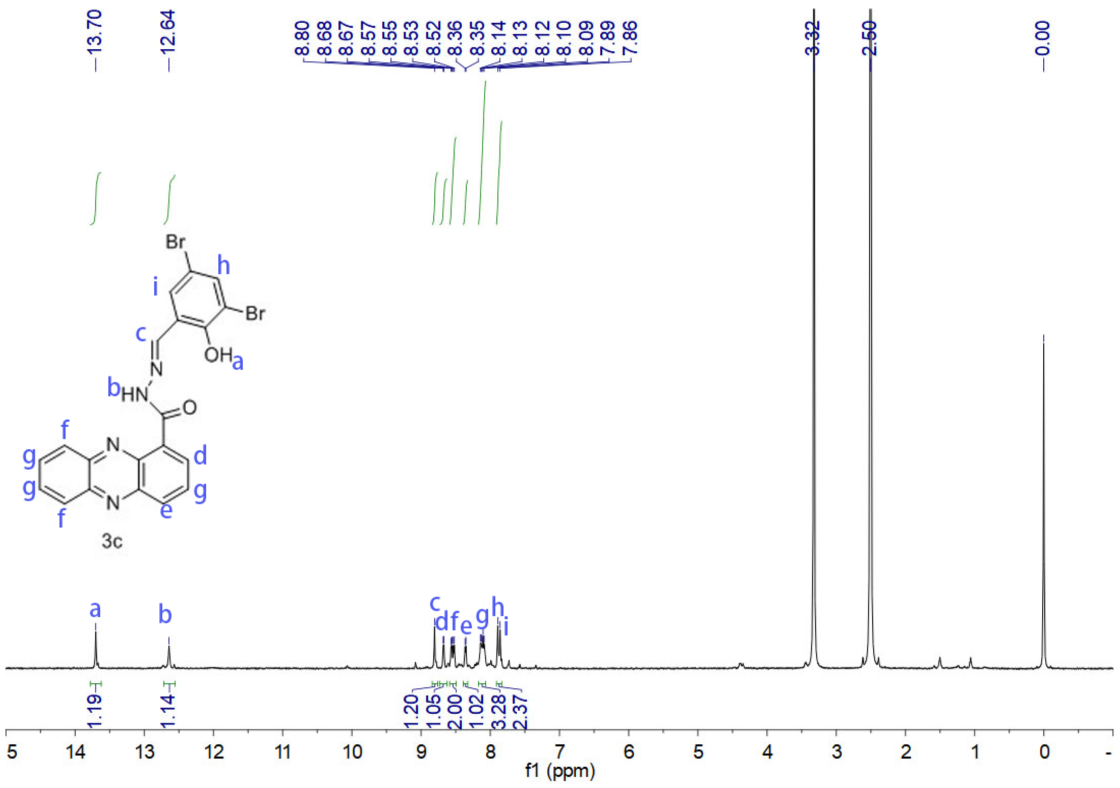

Figure S3. ^{1}H NMR of compound **3c**

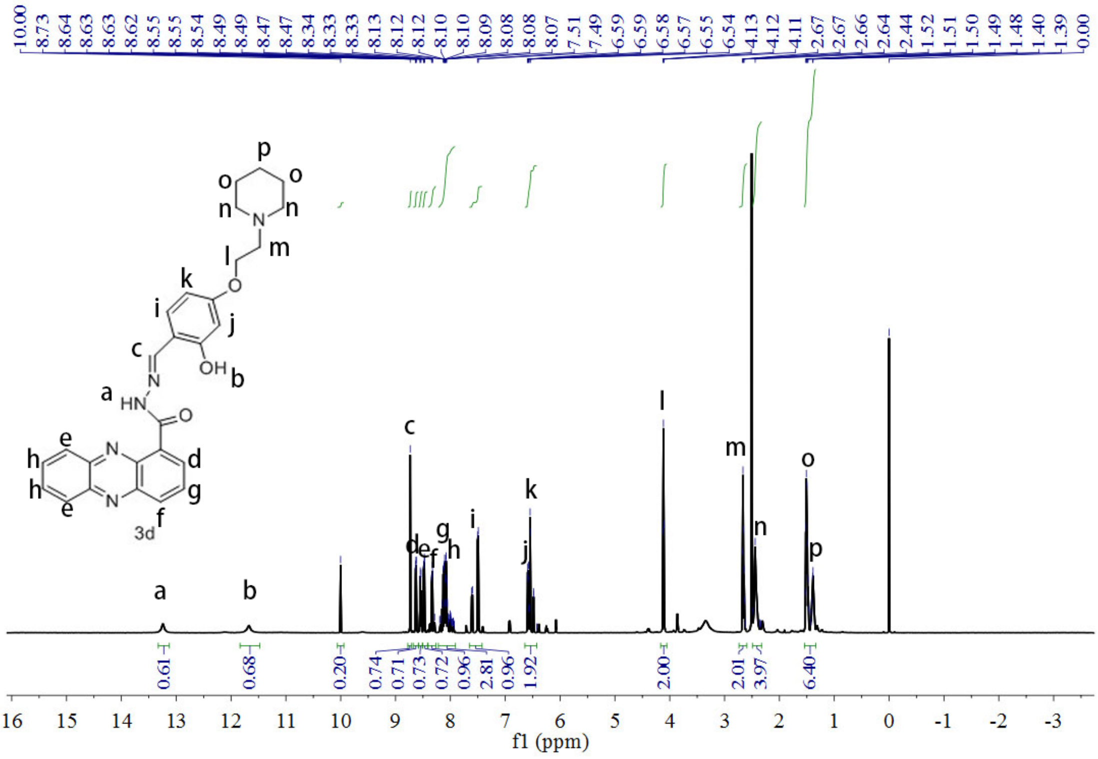

Figure S4. ^{1}H NMR of compound **3d**

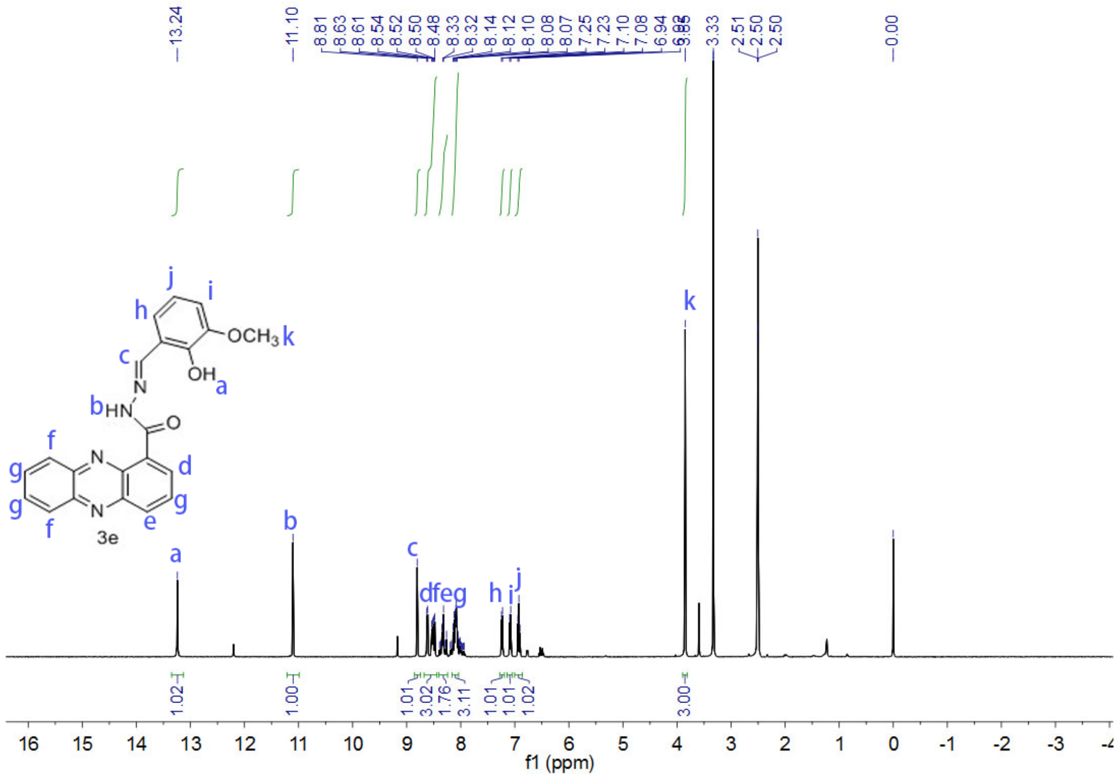

Figure S5. ^{1}H NMR of compound **3e**

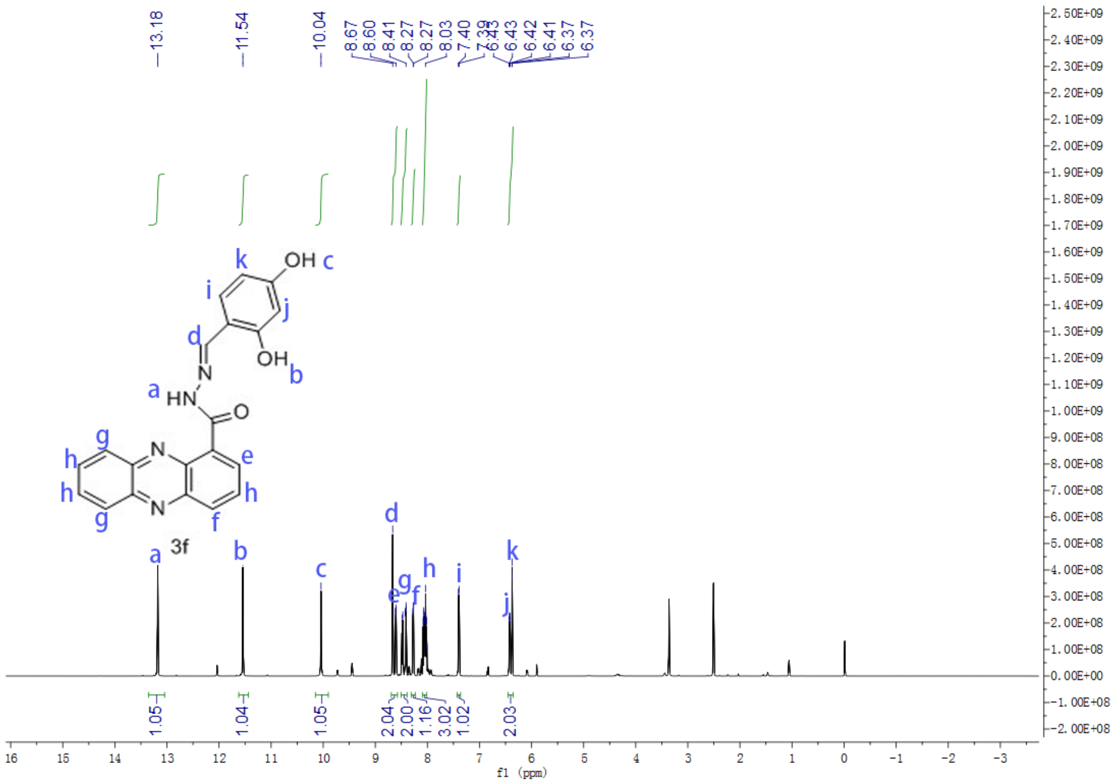

Figure S6. ^{1}H NMR of compound **3f**

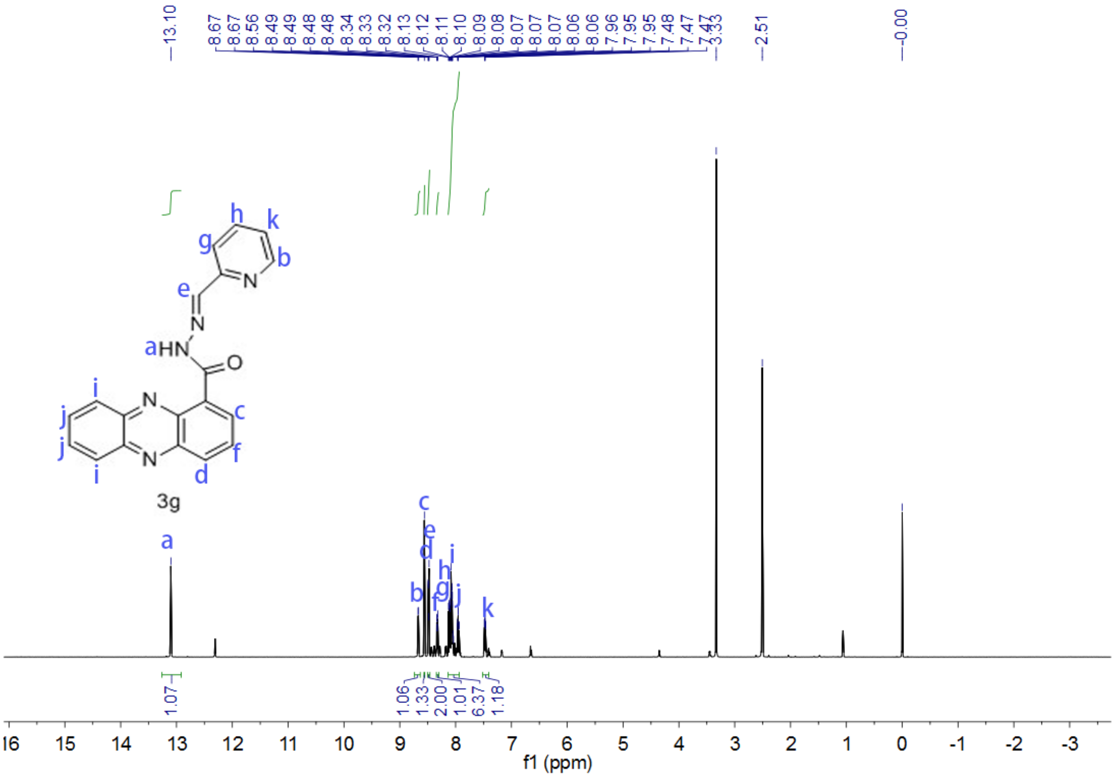

Figure S7. ^{1}H NMR of compound **3g**

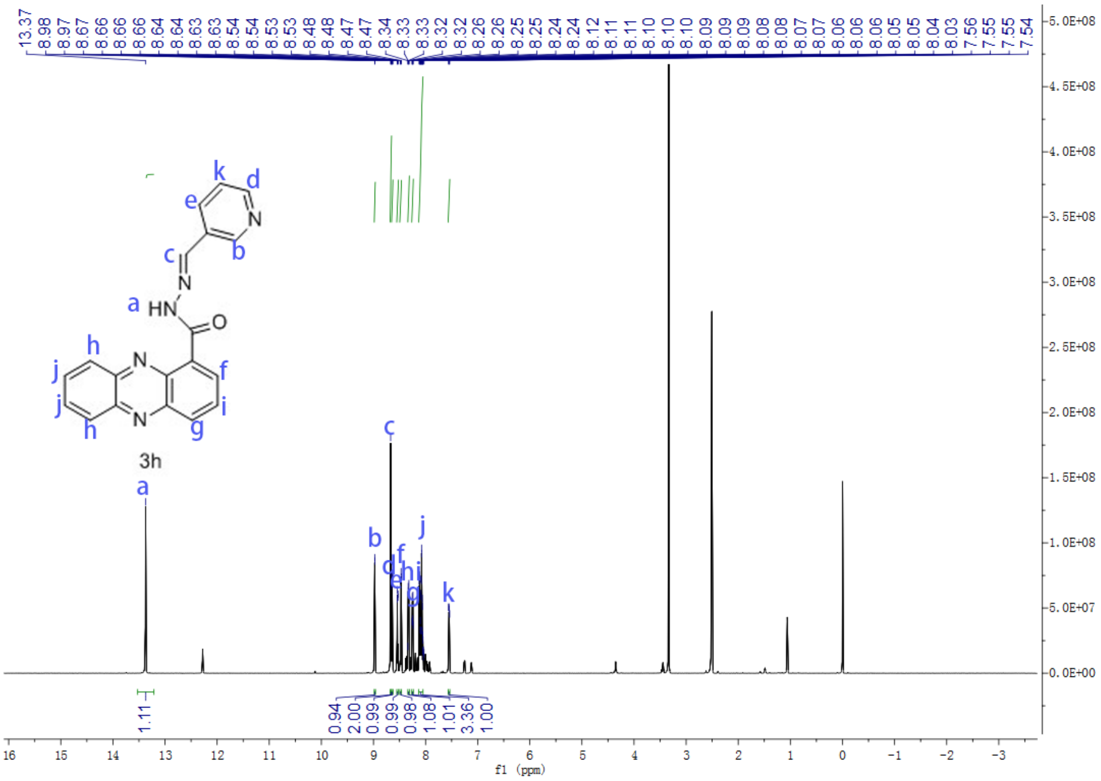

Figure S8. ^{1}H NMR of compound **3h**

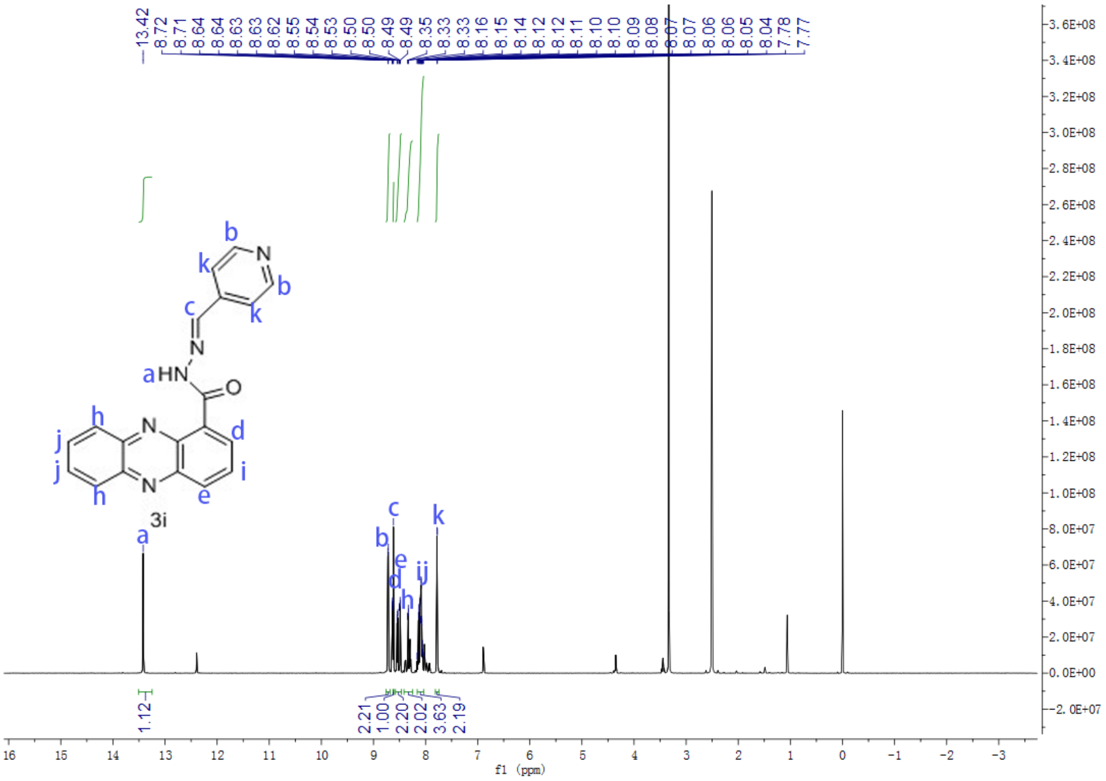

Figure S9. ^{1}H NMR of compound **3i**

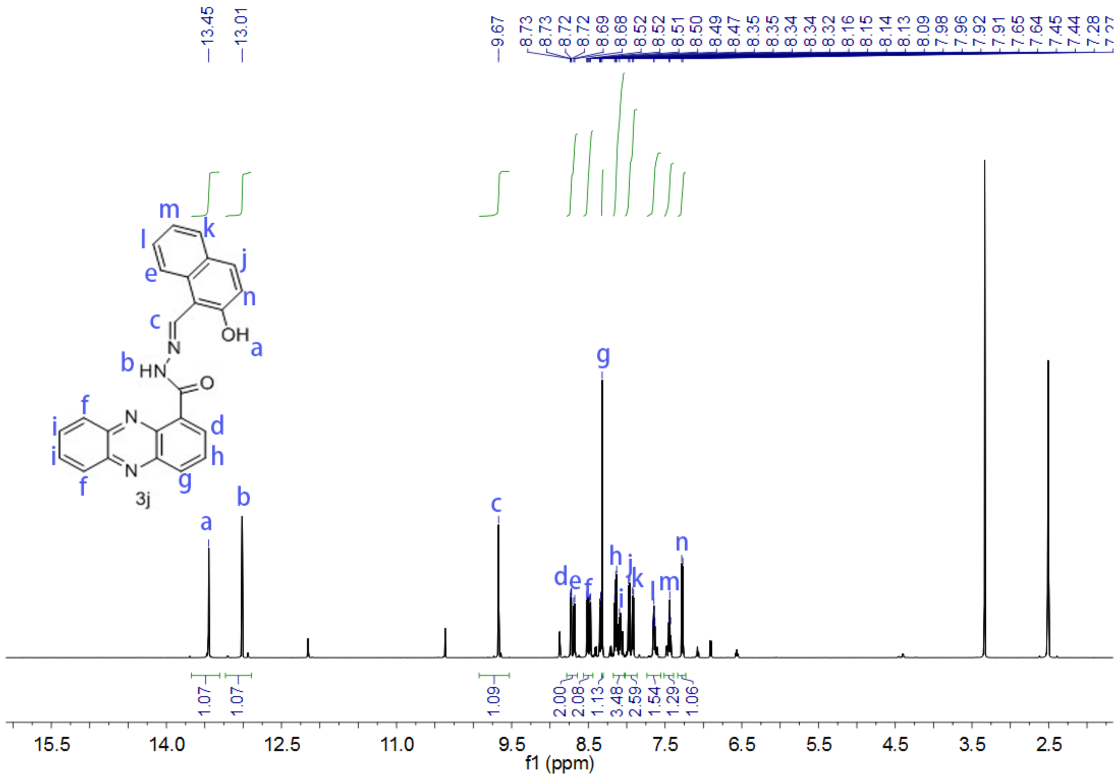

Figure S10. ^{1}H NMR of compound 3j

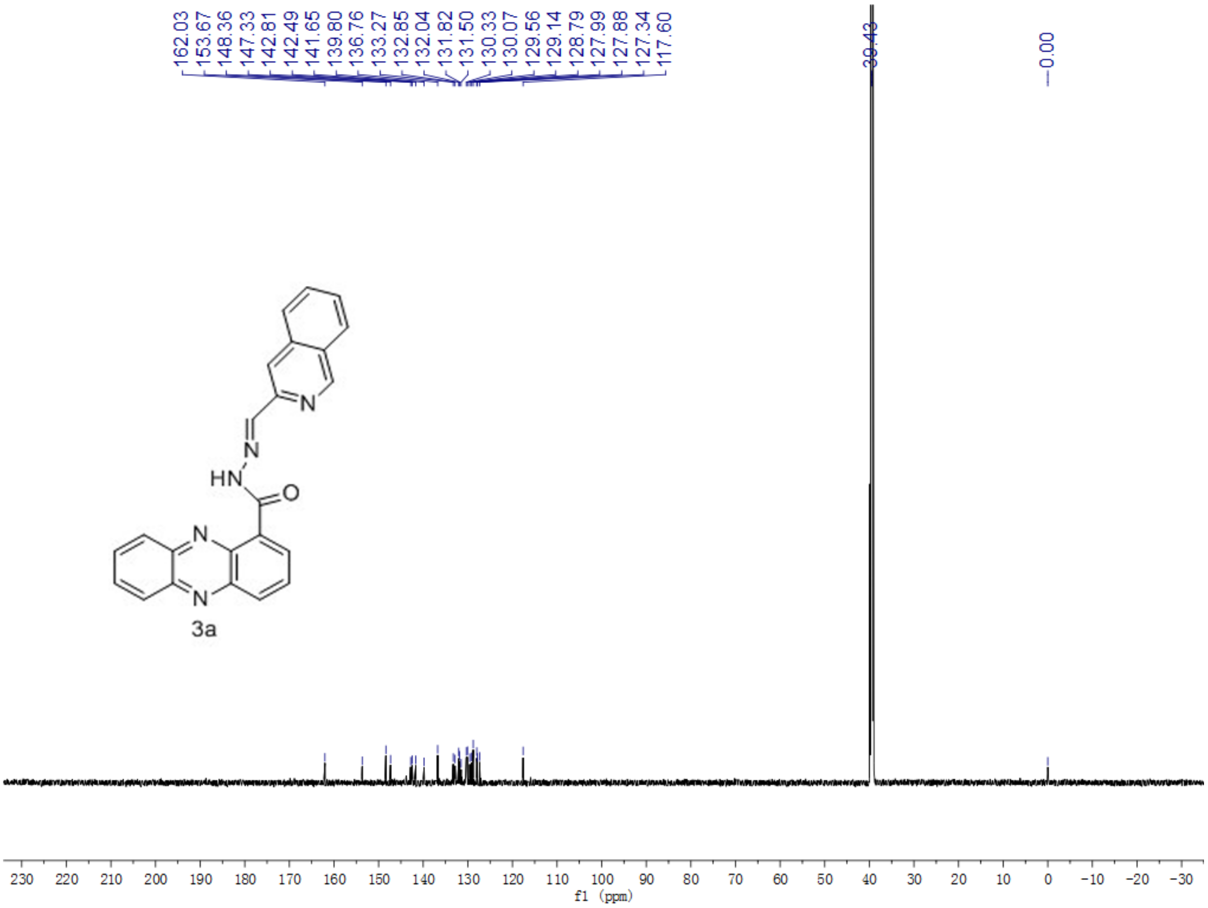

Figure S11. ^{13}C NMR of compound **3a**

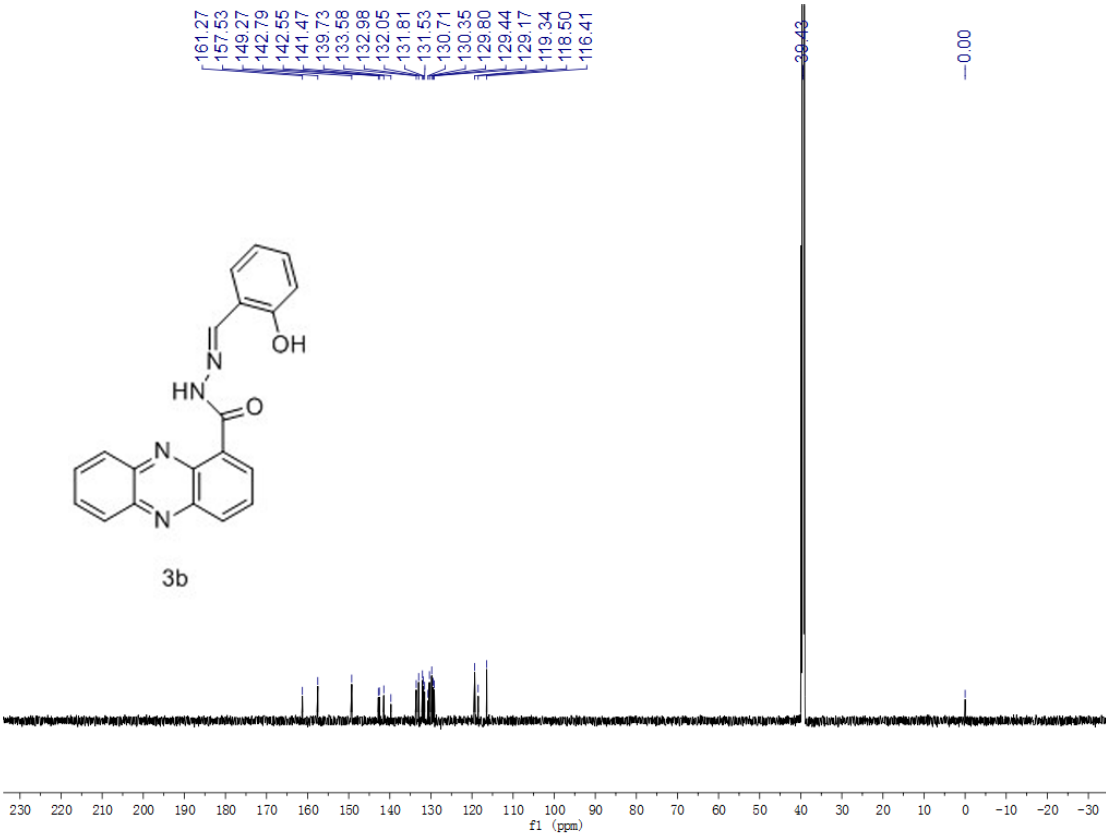

Figure S12. ^{13}C NMR of compound **3b**

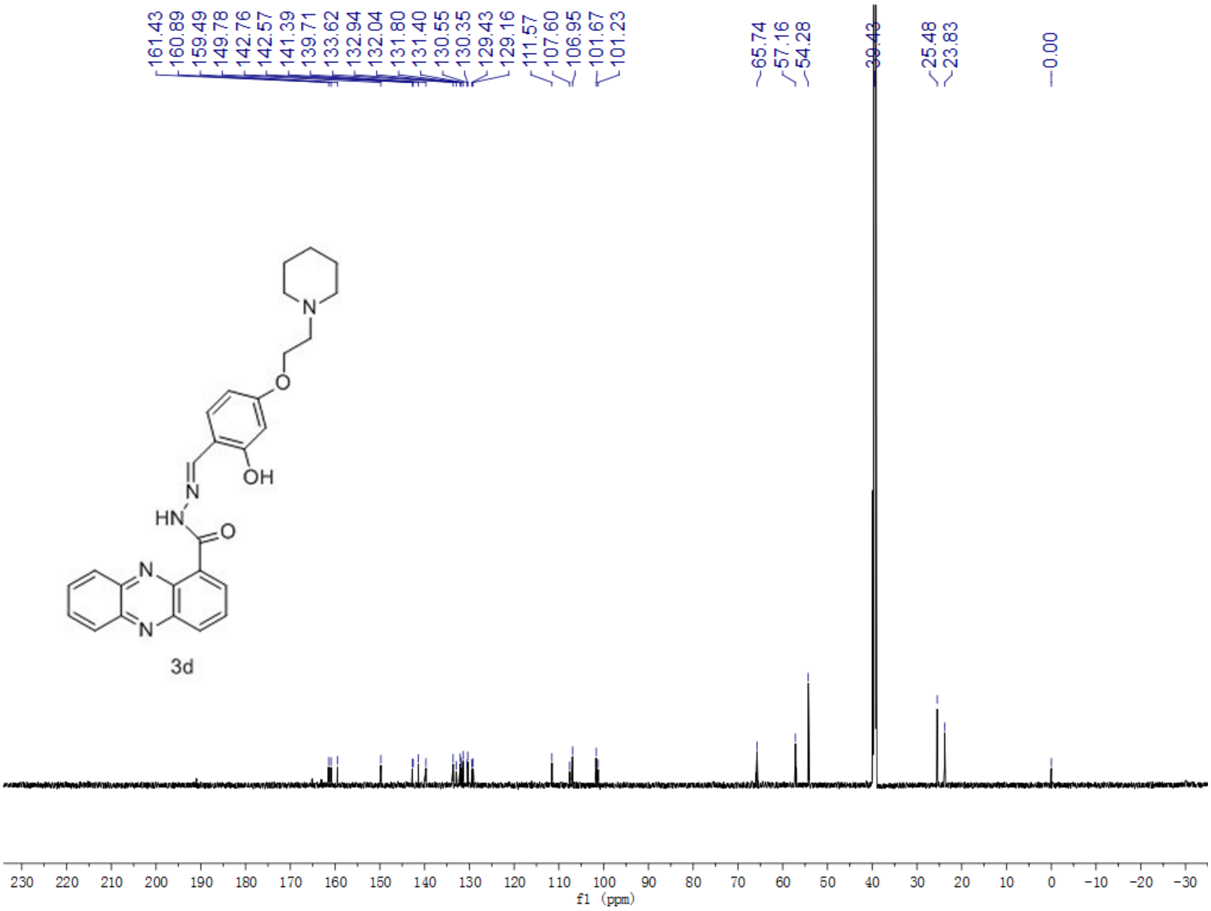

Figure S13. ^{13}C NMR of compound **3d**

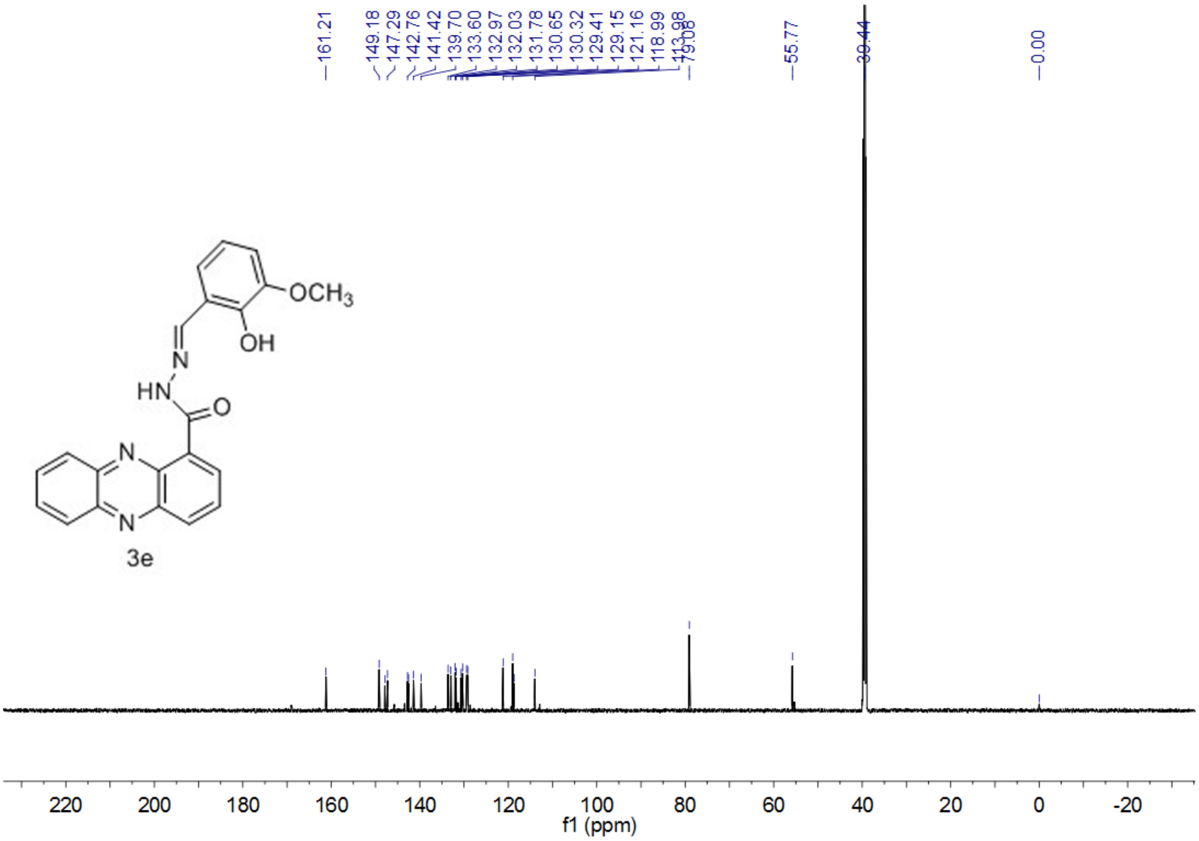

Figure S14. ^{13}C NMR of compound **3e**

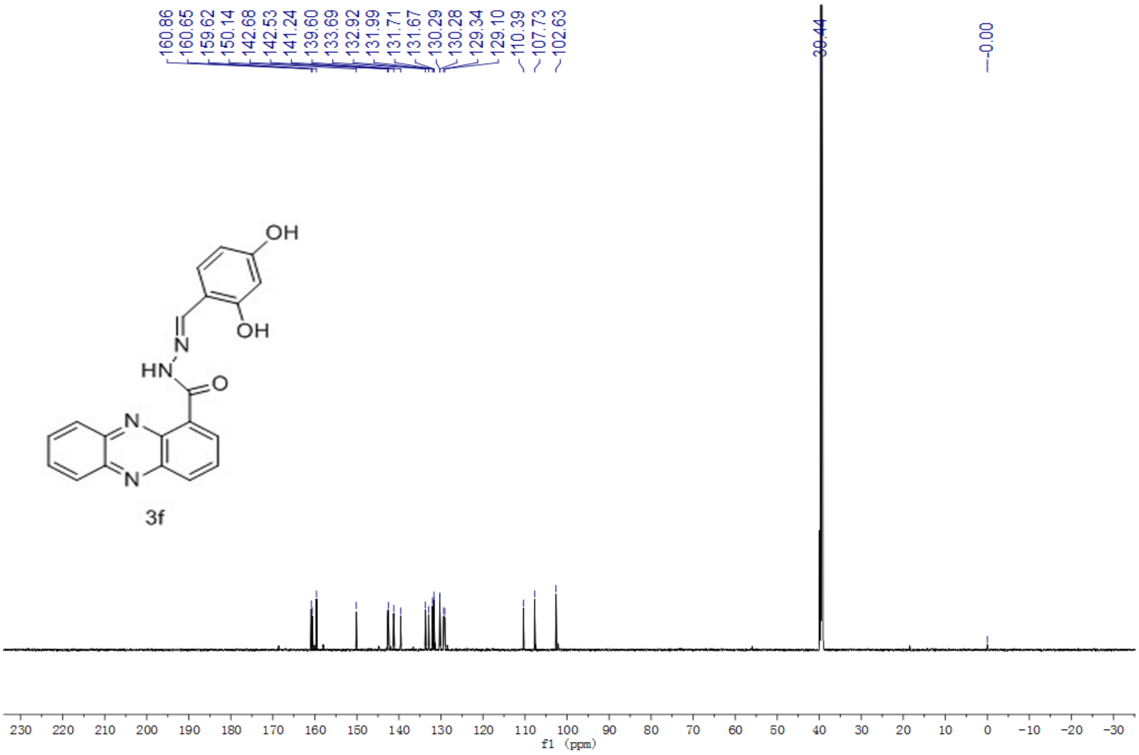

Figure S15. ^{13}C NMR of compound **3f**

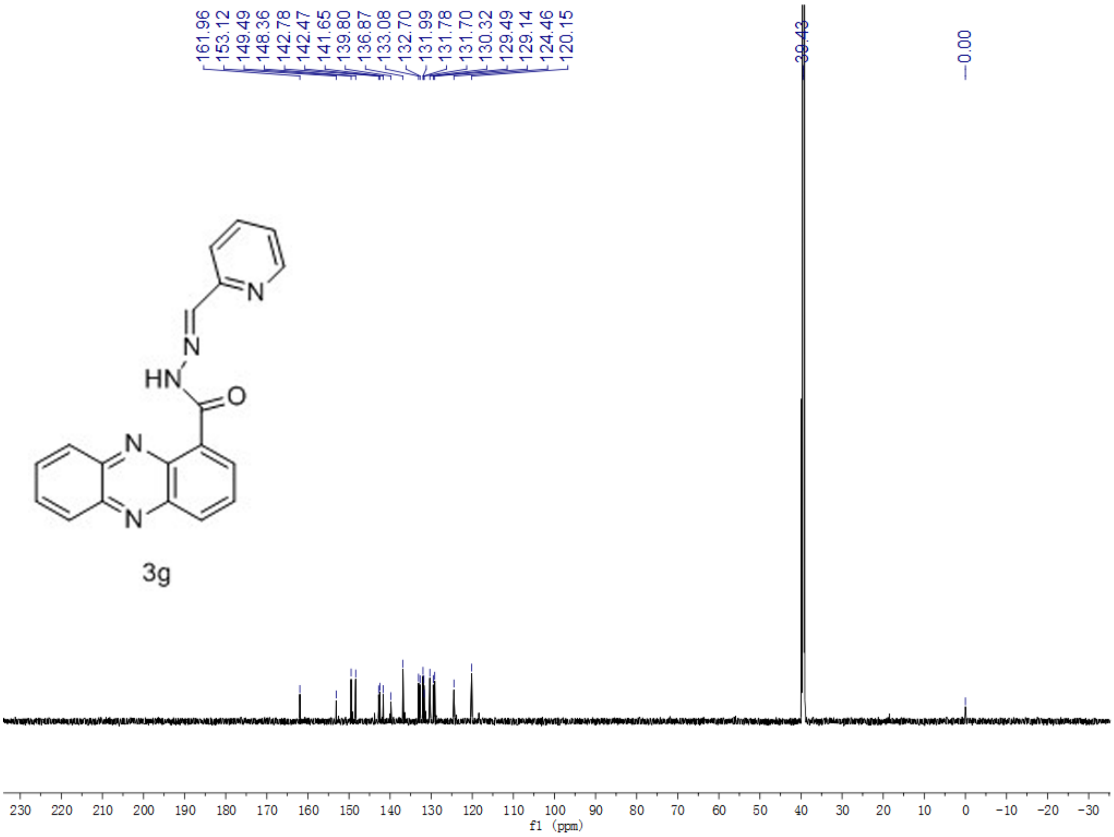

Figure S16. ^{13}C NMR of compound **3g**

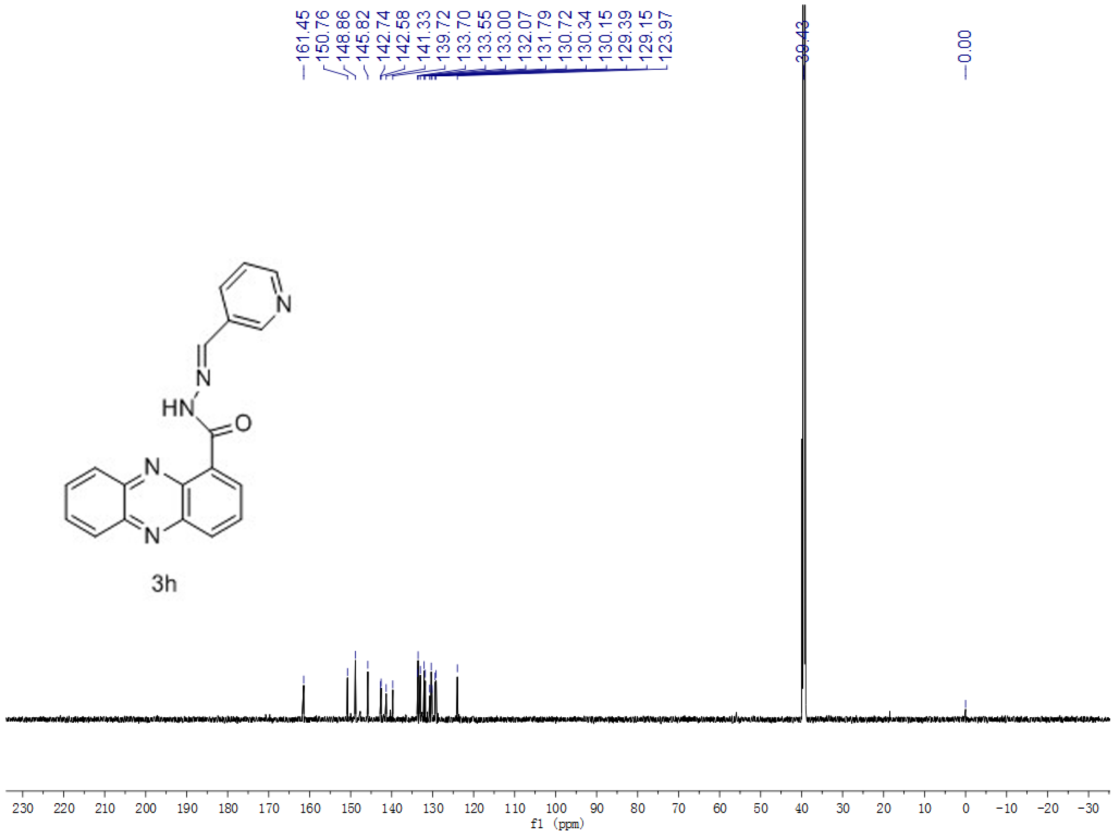

Figure S17. ^{13}C NMR of compound **3h**

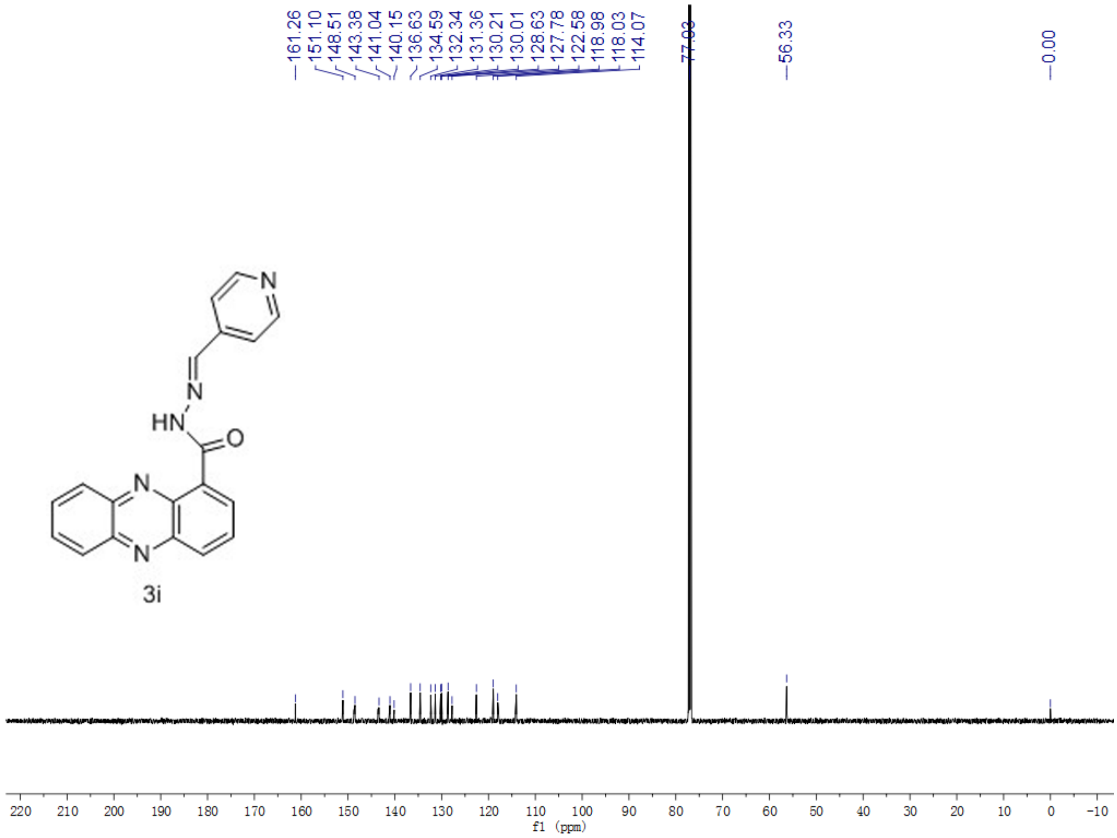

Figure S18. ^{13}C NMR of compound **3i**

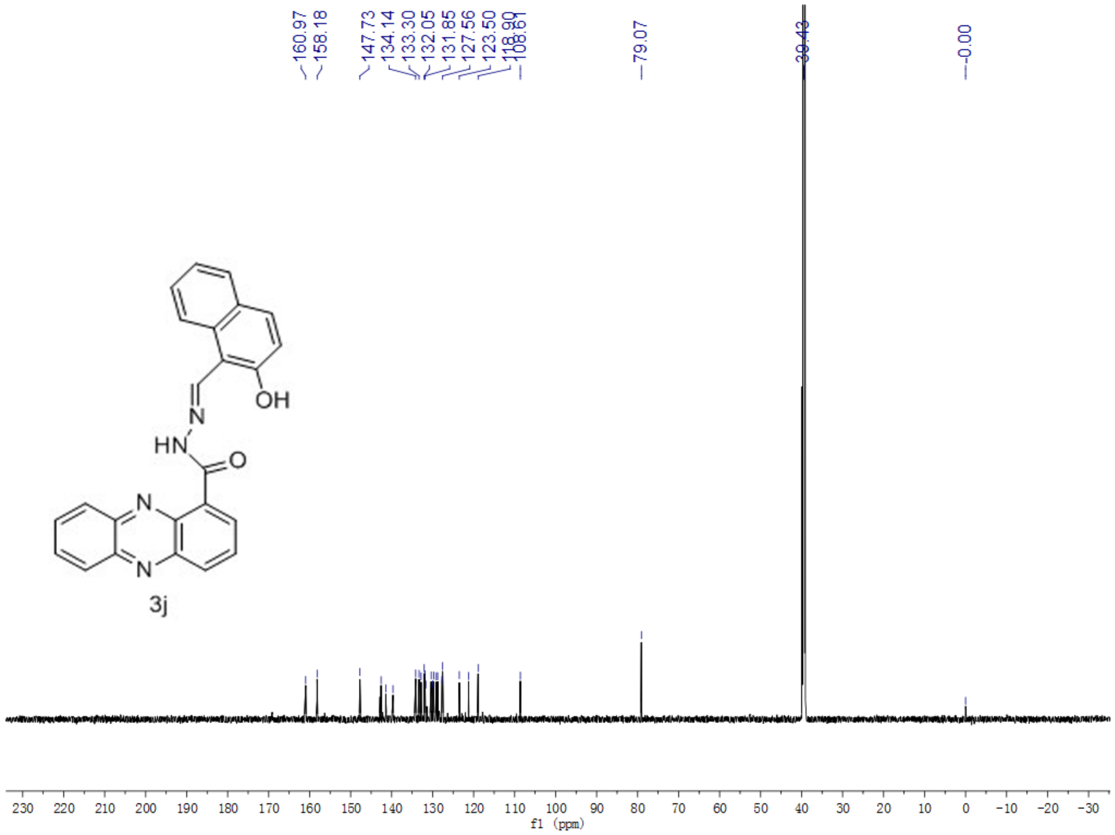

Figure S19. ^{13}C NMR of compound 3j

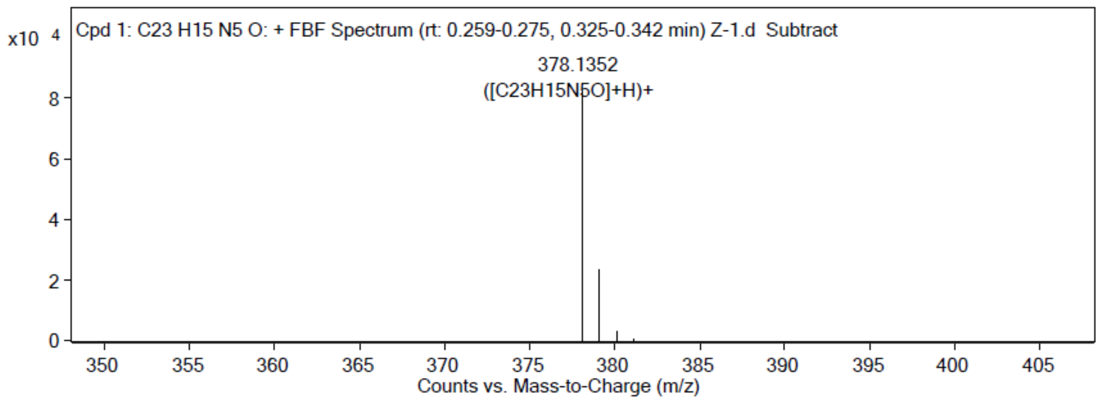

FigureS20. HRMS of compounds 3a

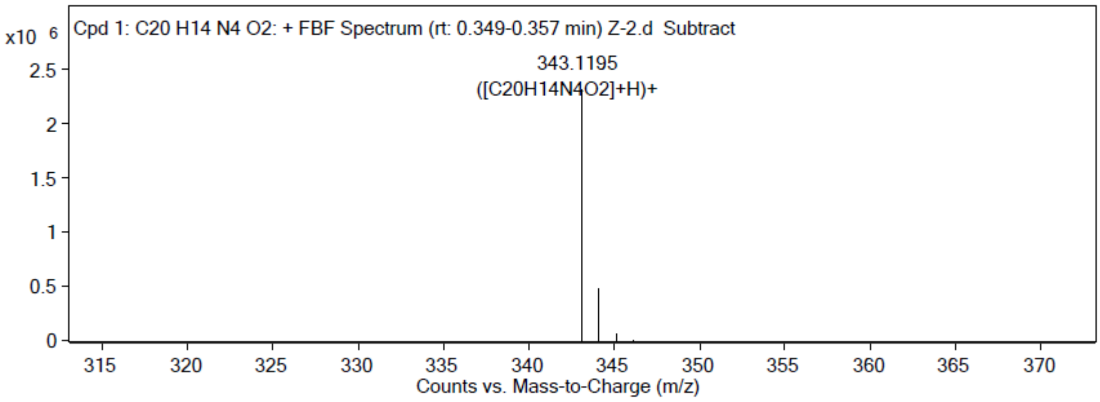

FigureS21. HRMS of compounds 3b

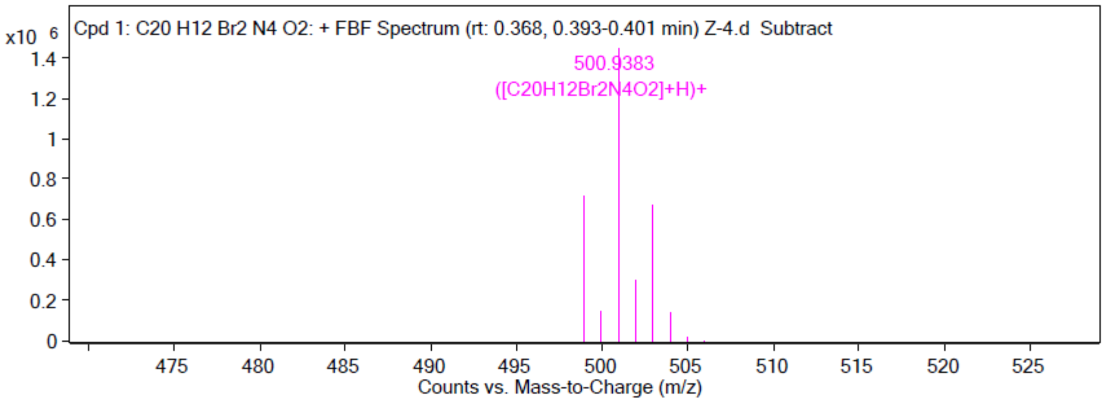

FigureS22. HRMS of compounds 3c

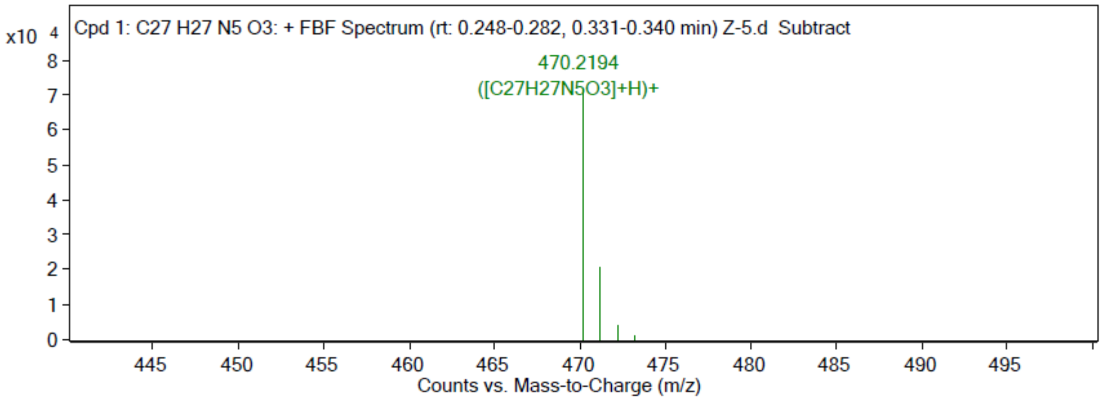

FigureS23. HRMS of compounds 3d

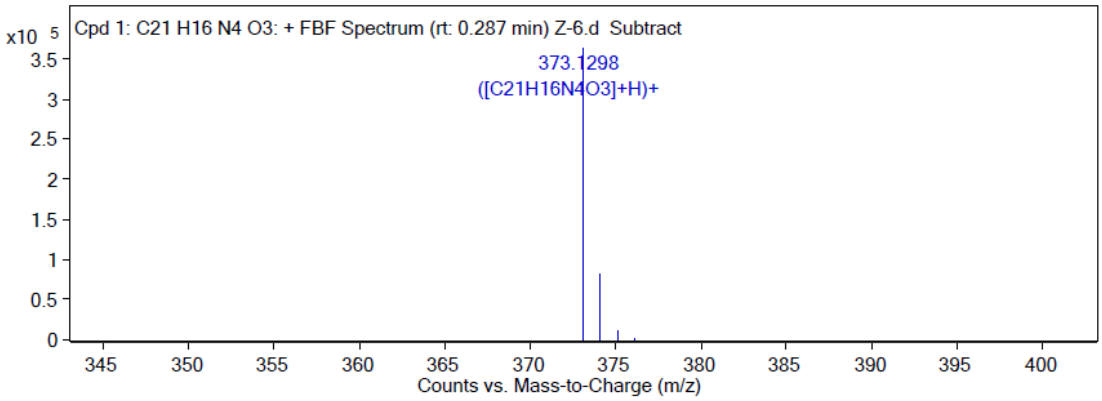

FigureS24. HRMS of compounds 3e

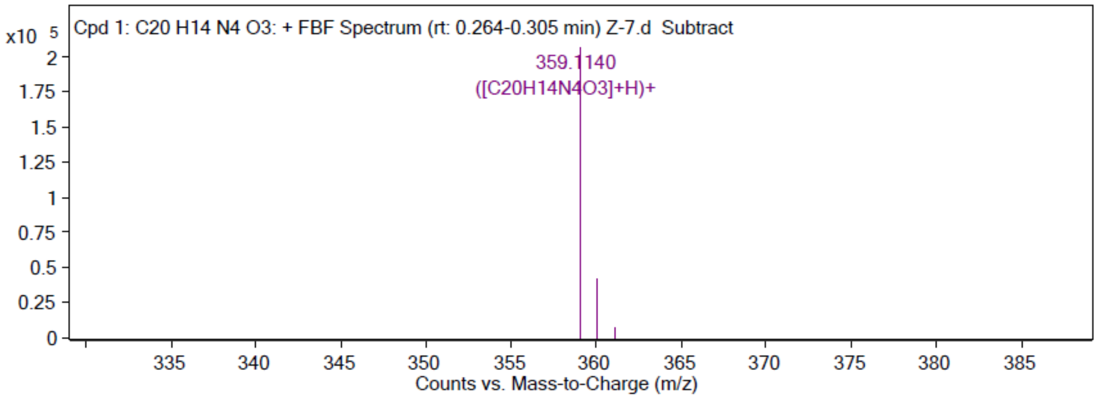

FigureS25. HRMS of compounds 3

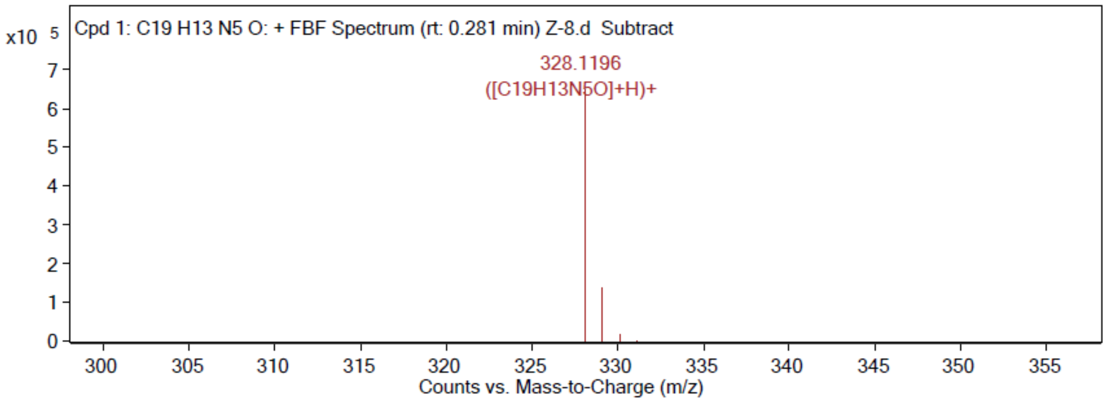

FigureS26. HRMS of compounds 3g

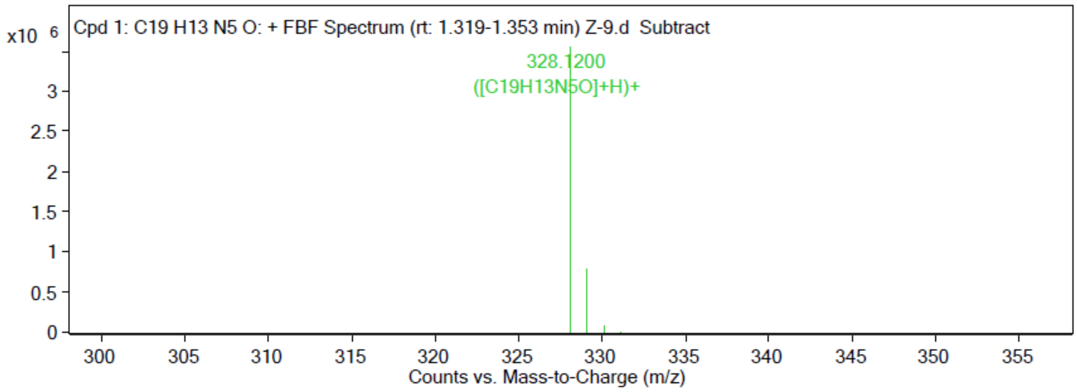

FigureS27. HRMS of compounds 3h

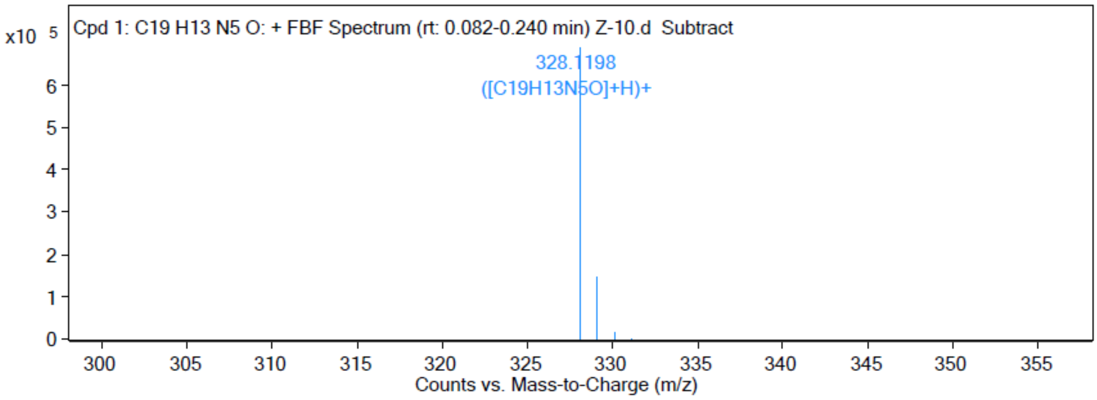

FigureS28. HRMS of compounds 3i

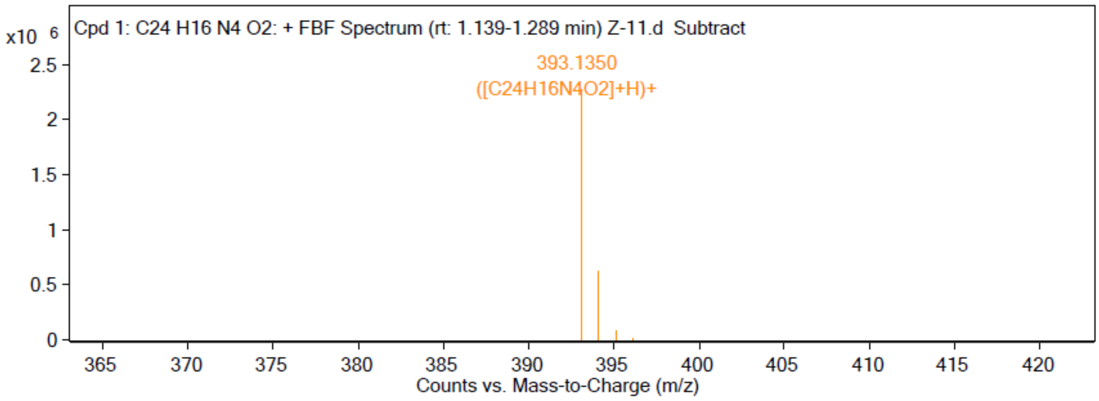

FigureS29. HRMS of compounds 3j

Table S1. Crystal data and structure refinement for the compound 3a~3c

	**3a**	**3b**	**3c**
Empirical formula	$C_{24}H_{16}Cl_3N_5O$	$C_{20}H_{14}N_4O_2$	$C_{23}H_{19}Br_2N_5O_3$
Formula weight	496.77	342.35	573.25
Temperature / K	293(2)	296(2)	293(2)
Crystal system	Triclinic	Monoclinic	Monoclinic
Space group	*P*-1	$P2_1/n$	$P2_1/n$
a / nm	0.7133(10)	0.63479(19)	0.42714(4)
b / nm	0.9190(13)	1.1742(3)	2.06455(19)
c / nm	1.884(3)	2.2504(6)	2.7607(3)
α/(°)	81.52(2)	90.00	90.00
β/(°)	87.70(3)	94.049(9)	91.200(3)
γ(°)	74.49(3)	90.00	90.00
V / nm^3	1.177(3)	1.6732(8)	2.4340(4)
Z	2	4	4
D_c / (g·cm^{-3})	1.402	1.359	1.564
μ/mm^{-1}	0.416	0.091	3.364
F(000)	508	712	1144
Crystal size /mm	0.15 × 0.12 × 0.09	0.25 × 0.24 × 0.23	0.22 × 0.21 × 0.18
Reflection	3980	10656	15479
Unique	3329	2948	4261
Observed reflection	1288	1893	2853
Number of parameters	613	235	298
GOF	1.246	1.012	1.066
Final *R* indices [I >2σ(I)]	R_1= 0.2464 , wR_2= 0.6126	R_1= 0.0452, wR_2= 0.1225	R_1= 0.0622, wR_2= 0.2105
R indices (all data)	R_1= 0.3781 , wR_2= 0.6606	R_1= 0.0764, wR_2= 0.1491	R_1= 0.0971, wR_2= 0.2355

Table S2. Crystal data and structure refinement for the compound 3e~3g

	**3e**	**3f**	**3g**
Empirical formula	$C_{20}H_{20}N_2O_2$	$C_{20}H_{16}N_{3.50}O_4$	$C_{19}H_{13}N_5O$
Formula weight	320.38	369.36	327.34
Temperature / K	293(2)	296(2)	293(2)
Crystal system	Triclinic	Monoclinic	Monoclinic
Space group	*C2/c*	$P2_1/c$	$P2_1/n$
a / nm	4.5832(6)	0.61094(4)	0.72472(8)
b / nm	0.77111(10)	1.15598(7)	0.51293(5)
c / nm	2.9470(4)	2.50951(16)	2.0804(2)
α/(°)	90.00	90.00	90.00
β/(°)	129.043(3)	92.401(2)	95.915(3)
γ(°)	90.00	90.00	90.00
V / nm^3	8.0893(18)	1.77075(19)	7.6922(14)
Z	18	4	2
D_c / (g·cm^{-3})	1.184	1.385	1.413
μ/mm^{-1}	0.077	0.099	0.093
F(000)	3060	770	340
Crystal size /mm	0.21 × 0.20 × 0.19	0.24 × 0.23 × 0.21	0.21 × 0.20 × 0.18
Reflection	25327	11178	4585
Unique	7127	3106	2159
Observed reflection	3613	2148	1834
Number of parameters	554	256	226
GOF	1.036	1.031	1.030
Final *R* indices [$I > 2\sigma(I)$]	R_1= 0.0635, wR_2= 0.1780	R_1= 0.0391, wR_2= 0.0949	R_1= 0.0365, wR_2= 0.0797
R indices (all data)	R_1= 0.1265, wR_2= 0.2296	R_1= 0.0623, wR_2= 0.1109	R_1= 0.0461, wR_2= 0.0859

Table S3. Crystal data and structure refinement for the compound 3h~3k

	**3h**	**3i**	**3k**
Empirical formula	$C_{19}H_{13}N_5O$	$C_{19}H_{13}N_5O$	$C_{24}H_{16}N_4O_2$
Formula weight	327.34	327.34	392.41
Temperature / K	293(2)	296(2)	293(2)
Crystal system	Orthorhombic	Monoclinic	Monoclinic
Space group	$Pna2_1$	$P2_1/c$	$P2_1/n$
a / nm	2.4707(5)	1.8601(5)	0.76260(17)
b / nm	0.61064(13)	1.1099(5)	1.3156(3)
c / nm	2.0947(4)	1.5098(5)	1.8946(4)
α/(°)	90.00	90.00	90.00
β/(°)	90.00	94.077(5)	98.776(7)
γ(°)	90.00	90.00	90.00
V / nm^3	3.1603(12)	3.1091(19)	1.8786(7)
Z	8	8	4
D_c / (g·cm^{-3})	1.376	1.399	1.387
μ/mm^{-1}	0.090	0.092	0.091
F(000)	1360	1360	816
Crystal size /mm	0.23 × 0.21 × 0.21	0.23 × 0.21 × 0.20	0.22 × 0.21 × 0.18
Reflection	9929	19821	11916
Unique	3617	5481	3302
Observed reflection	1779	3543	1486
Number of parameters	451	452	271
GOF	0.970	1.012	1.012
Final R indices [I >2σ(I)]	R_1= 0.0574, wR_2= 0.1101	R_1= 0.0480, wR_2= 0.1106	R_1= 0.0614, wR_2= 0.1669
R indices (all data)	R_1= 0.1436, wR_2= 0.1491	R_1= 0.0825, wR_2= 0.1331	R_1= 0.1405, wR_2= 0.2175

Table S4. Hydrogen bond parameters [Å and °] in the crystal structure of compounds 3a-3j

D−H---A	d(D−H) (Å)	d(H---A) (Å)	d(D---A) (Å)	∠(DHA) (°)
		**3a**		
N3−H3---N4	0.86	2.09	2.775(18)	136
C5−H5---O1	0.93	2.50	3.34(2)	151
C7−H7---O1	0.93	2.57	3.39(2)	146
C13−H13---O1	0.93	2.41	2.76(2)	102
C47−H47---N5	0.98	2.18	3.14(3)	167
		**3b**		
O2−H2A---N4	0.82	2.35	2.617(3)	100
N3−H3A---N2	0.86	1.99	2.686(3)	137
C10−H10---O1	0.93	2.43	2.773(3)	102
		**3c**		
O2−H2A---N4	0.82	1.88	2.598(8)	145
N3−H3A---N2	0.86	1.94	2.658(8)	140
C10−H10---O1	0.93	2.39	2.749(10)	103
C23−H23C---O2	0.96	2.28	3.226(17)	168
		**3e**		
O1W−H1WA---N5	0.85	2.05	2.895(4)	172
O1W−H1WB---O3	0.85	1.97	2.814(3)	172
O1−H2A---N4	0.82	1.88	2.594(3)	146
N3−H3A---N2	0.86	2.01	2.697(4)	136
O5−H5A---N8	0.82	1.93	2.644(2)	145
N7−H7---N6	0.86	1.96	2.669(3)	139
C3−H3---O5	0.93	2.57	3.466(4)	162
C10−H10---O5	0.93	2.40	2.727(5)	102
C14−H14---O1W	0.93	2.54	3.178(4)	126
C24−H24---O1	0.93	2.59	3.499(4)	166
C32−H32---O6	0.93	2.39	2.744(3)	102
		**3f**		
N1−H1---N3	0.86	1.99	2.6746(18)	136
O1W−H1WA---O3	0.85	2.03	2.854(2)	164
O1−H1A---O1W	0.82	1.85	2.672(2)	174
O1W−H1WB---O2	0.85	1.99	2.834(2)	176
O2−H2A---N3	0.83	1.84	2.5625(18)	147
C10−H10---O3	0.93	2.41	2.755(2)	101
		**3g**		
N3−H3A---O002	0.86	2.16	2.973(3)	158
N3−H3A---N1	0.86	2.42	2.876(4)	114
C11−H11---N5'	0.93	2.50	3.417(5)	168
		**3h**		
N3−H3A---N1	0.86	1.99	2.691(9)	138
N7−H7---N5	0.86	1.99	2.688(9)	137
C10−H10---O2	0.93	2.40	2.742(11)	101
C17'−H17'---O1	0.93	2.60	3.355(11)	139

C20−H20···O1	0.93	2.36	2.728(11)	103
C26−H26···O1	0.93	2.45	3.122(12)	129
C38−H38···N10	0.93	2.57	3.417(12)	151
		**3i**		
N3−H3A···N2	0.86	2.04	2.720(3)	135
N8−H8A···N7	0.86	1.96	2.673(3)	139
C8−H8···N6	0.93	2.58	3.513(3)	177
C10−H10···O1	0.93	2.38	2.733(3)	102
C23−H23···O2	0.93	2.40	2.752(2)	102
		**3j**		
O2−H2A···N4	0.82	1.92	2.576(4)	136
N3−H3A···N2	0.86	1.97	2.669(4)	138
C10−H10···O1	0.93	2.41	2.752(5)	102
C17−H17···O1	0.93	2.53	3.457(6)	175
